# Comprehensive Review on Monitoring, Behavior, and Impact of Pesticide Residues during Beer-Making

**DOI:** 10.1021/acs.jafc.2c07830

**Published:** 2023-01-18

**Authors:** Gabriel Pérez-Lucas, Ginés Navarro, Simón Navarro

**Affiliations:** Department of Agricultural Chemistry, Geology and Pedology, School of Chemistry, University of Murcia, Campus Universitario de Espinardo, E-30100 Murcia, Spain

**Keywords:** brewing, quality control, plant protection products, toxicological risk

## Abstract

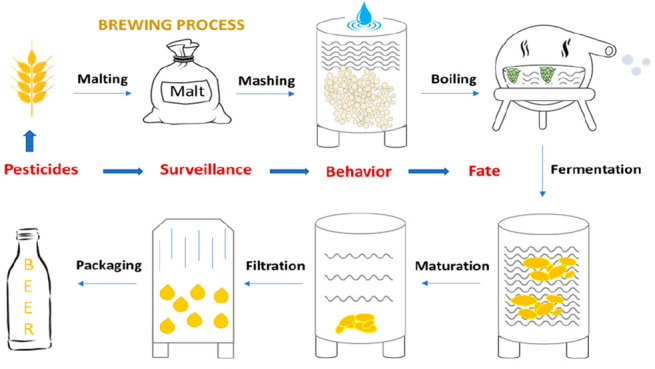

This paper reviews the impact of beer-making stages (malting, mashing, boiling, and fermentation) on the behavior of pesticide residues. The large use of pesticides on barley and hop could cause the occurrence of their residues in beer. The foremost factors influencing the stability of residues (pH, temperature, and water content) and the physical-chemical properties of pesticides (octanol–water partition coefficient, vapor pressure, and water solubility) are essential to know their final fate. Most pesticides show a decrease in the unhopped wort because they are adsorbed onto the spent grains after mashing. In addition, their concentrations decrease during boiling and fermentation. Generally, maltsters should dedicate particular attention to the residues of hydrophobic pesticides because they can remain on the malt. Contrarily, brewers should control residues of hydrophilic pesticides because they can be carried over into young beer, disturbing the quality and organoleptic properties (flavor, aroma, taste, or color) of the beer.

## 1 Introduction

The yield of many crops can be severely reduced due to numerous and different pests and diseases.^1^ To defend crops (before and after harvest), different pesticide classes are usually applied by farmers to fight pests and diseases.^2,3^ The application of pesticides in agriculture enhance the yield, improve the quality as well as expand the storage life of food crops. They are usually used to guarantee effective fruit and vegetable production.^4^ However, their often large-spectrum biocide activity and potential risk to the consumers are a growing source of concern for the population and environment.^5,6^ A pesticide also called plant protection product (PPP) includes substances such as insecticides, fungicides, and herbicides among other minor groups.^7,8^ Regulation (EC) No 1107/2009^9^ is the legislation concerning the placing of PPPs on the market in the European Union (EU). The European Food Safety Agency (EFSA) Pesticides Unit in close cooperation with all 27-EU member states is the organization responsible for the EU of risk assessments (direct or indirect harmful consequences on human or animal health) of active substances present in PPPs.^10,11^ A probable effect of incorrect use may be their presence in the treated products after harvesting. Customers are unprotected to pesticides because small amounts can remain as residues in postharvest products. The residual amount found in foods must be as low as possible to safeguard the health of consumers, by corresponding to the lowest amount of pesticide used on the crop to achieve the desired effect. It is essential to guarantee that such residues should not be found in foods/feeds at levels representing an unacceptable risk to humans and animals.^12^

Pesticide residues in foods are influenced by the storage, handling, and processing occurring between harvesting of the raw agricultural commodities (RAC) and ingestion of processed foodstuffs.^13,14^ To evaluate the residue of PPPs, processing studies are important to better estimate the exposure of customers to residues.^15−18^ The results obtained allowed for a more realistic calculation of consumers’ intake of the active ingredients present in PPPs and/or their relevant transformation products and, consequently, a better risk assessment than that calculated from the theoretical maximum daily intake (TMDI). In addition, these studies can generate results relating to residues in commodities that may be used as animal feed stuffs. The foremost factors motivating the permanency of residues during food processing are pH, temperature, and water content as well as the chemical nature of the residue. Hydrolysis is most likely to disturb the nature of residues during food processing due to the fact that some processes such as heating can commonly inactivate enzymes existing on the substrate, leaving simple hydrolysis as main degradation route.^19^

**Figure 1:**
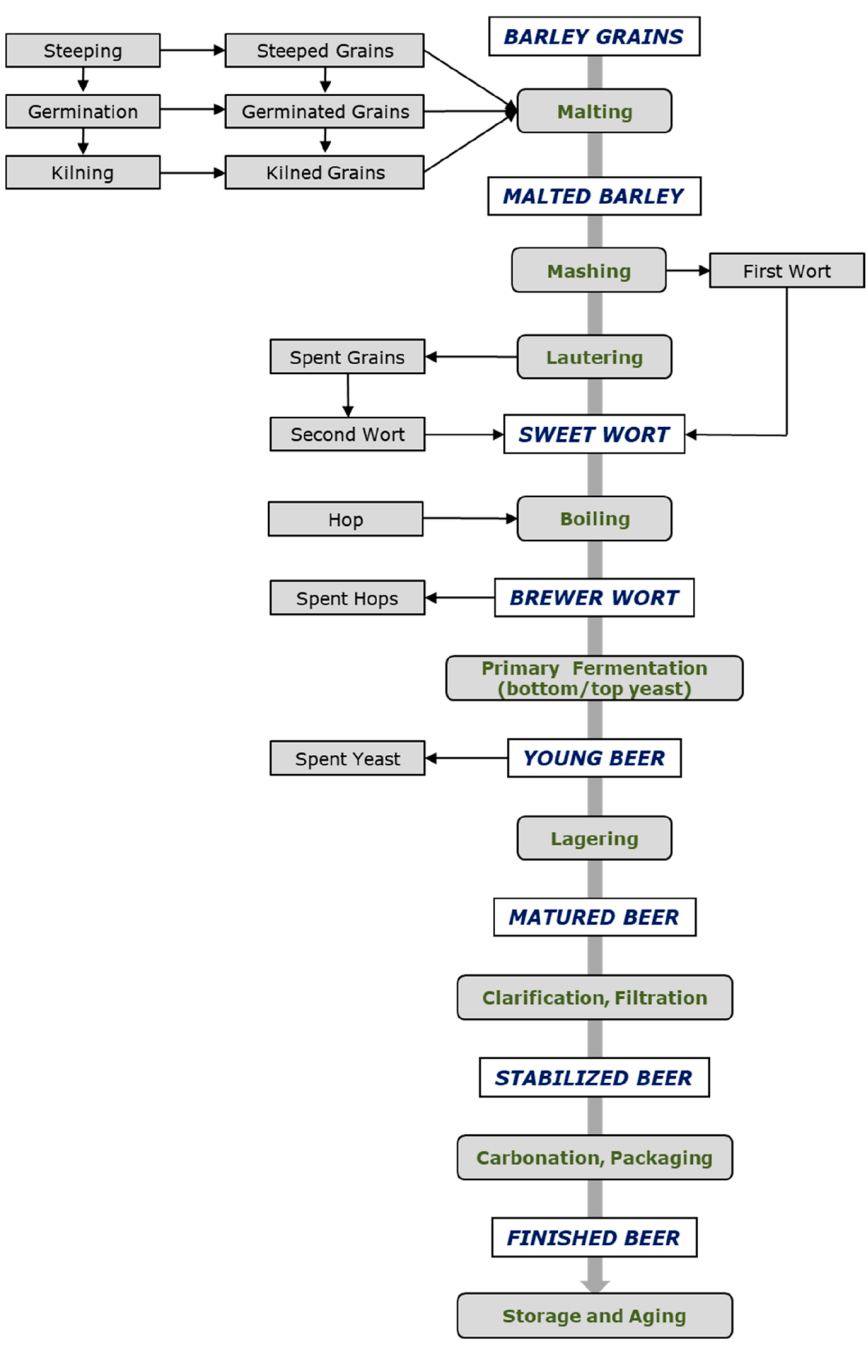
Scheme showing the main stages of the brewing process.^50^

Alcoholic beverages, beers, wines, and spirits (distilled beverages such as whisky, rum, gin, vodka, etc.) have long been an inseparable part of human societies, and its cultural, societal, and ritualistic importance cannot be overstated.^20^ Concretely, barley, hop, water, and yeasts are the main ingredients for beer-making. Beer can be defined as “a beverage produced by alcoholic fermentation of barley or wheat malt with hop in water, carried out by either brewer’s yeast or a mixture of yeast and other microbes”. Barley is the most used cereal for malting, but malt can be also obtained from rye, wheat, and other cereals.^21^Figure 1 shows a scheme of the malting and brewing processes, briefly described below.

### Malting

The process of converting barley to malt is: barley drying and storage, steeping, germination, and kilning. The steeping of barley in water promotes the development of hydrolytic enzymes. Germination is targeted to produce the maximum available extractable material through enzymatic activity. Finally, the “green malt” is kilned to detain germination and stabilize the malt by lowering moisture levels (<5%).

- *Milling*: Grinding the malt.
- *Mashing*: Mixing grist with water.
- *Wort separation*: Separating the liquid (wort) from the solid (spent grains) by lautering and mash filtration.
- *Wort boiling*: Sterilization, coagulation, hop extraction, and concentration.
- *Trub removal*: Removing coagulated material and hop debris (centrifugation, sedimentation filtration, and whirlpool.
- *Wort cooling/aeration*: Aerate and cool the wort.
- *Yeast handling*: Yeast propagation and storage and acid washing to reduce bacteria.
- *Yeast pitching*: Adding the culture yeast to the brewer wort.
- *Fermentation*: Yeast growth (C_2_H_6_O and CO_2_ generation).
- *Yeast removal*: Reduces yeast level in the young beer.
- *Aging*: Beer maturation at low temperature.
- *Clarification*: Particle removal to produce bright beer (finning, centrifugation and filtration).
- *Packaging*: Beer filling into final containers.
- *Warehousing and distribution*: Storing and transporting the beer to final costumer.

Currently there is a great interest highlighting the benefits of a moderate consumption of beer for human health, which is directly related to the absence of negative characteristics and the presence of positive attributes such as low sugar content and significant amounts of antioxidants, minerals, and vitamins.^22−24^ One of the main significant factors highlighting the public sensation of beer as a “healthy beverage” is the large number of studies demonstrating that moderate drinkers have lower death rates from all causes but specifically from cardiovascular-related diseases than either heavy drinkers or nondrinkers.^25^ The crop yield of malting barley is a very valuable factor in the malt production worldwide.^26^ During the growing season, various agrochemical sprays may be used to ensure the high quality and food safety of crop production. Pesticides remaining on a barley grain represent a potential source of unwanted pollution during beer-making. Additionally, to the optimal operation of the physiological functions of the barley being malted, a particular cause for concern is the potential health hazard from barley grains containing residues of pesticides, a problem that has afflicted the brewing industry in several countries. The quality of raw materials provides the basis for their handling and processing, and it has a decisive impact on the quality of beer.^27^ Therefore, the knowledge of the behavior and fate of pesticide residues during beer-making is an essential feature of the modern brewing industry. Hence, the objectives of this work are to review the occurrence and behavior of the pesticides commonly detected in barley and hop and their influence during beer-making stages (malting, mashing, boiling, and fermentation) to discuss their possible origin (sources) and fate.

## 2 Pests, Diseases, and Weeds on Barley and Hop

The cultivation of barley (*Hordeum vulgare*) and hop (*Humulus lupulus*) is commonly affected by different bacteria, fungus, virus, and pests. Barley grains represent a desirable source of nutrients for insects and microbial pathogens owing to their high content of starch and storage proteins. The exposure of the grain is increased during germination, when amino acids, fermentable carbohydrates, nitrogenous bases, and other degradation products of reserve polymers accumulate in the starchy endosperm.^28^ In consequence, several pests such as stem-borer, cutworms, armyworms, thrips or wheat aphids, and diseases like leaf spots, rusts, and powdery mildew (foliar diseases) or crown rot, covered smut, common root rot, black point, and root-lesion nematode (head and root diseases) can attack cereal crops, and a good weed (annual grasses and broad-leaved) control is indispensable if the crop is to make efficient use of moisture and to prevent weed seeds from polluting the harvest.^29^

Field insects are not generally a major hazard for barley crops, although significant damage can occur if conditions favoring the buildup of insect populations occur. Rotation development to minimize pest carryover, appropriate crop nutrition, and good control of weed and root diseases will all help in reducing the likelihood of damage by insect pests. Checking crops frequently throughout their growth for field insects and correctly identifying the insect pests is essential for their successful management. On the other hand, pests are not allowed in exported grains, thus the need for protecting the grain, which in turn saves money by not having grain rejected by a processor.

In addition, disease causing pathogens often decrease grain yield by damaging green leaves, preventing the production of sugars and proteins needed for growth. They can also block the plants internal transport mechanisms, reducing the movement of water and sugars through the plant. Yields are also reduced when the pathogen diverts the plants energy into producing more of the pathogen at the expense of plant growth or grain formation. The main pathogens that cause disease in barley are fungi, although viruses and nematodes can also damage crops. Furthermore, barley may be damaged by fungi (mainly by *Penicillium* and *Aspergillus* species) during storage, which generate secondary metabolites as ochratoxin A (OTA), characterized to be immunosuppressive and teratogenic. The International Agency for Research on Cancer (IARC) includes OTA (group 2B) as possibly carcinogenic to humans.^30^ Some of these metabolites cause gushing, a significant quality weakness where the beer spontaneously gushes from a bottle on opening. Therefore, it is very important to keep the storage of malting barley under optimal conditions to avoid fungal growth.^31^

Finally, weed competition can be impacted by crop species, crop variety, weed species, crop and weed density, and emergence time of the crop relative to the weed. Barley is more competitive with weeds than wheat, canola, and pulses when using at recommended seeding rates due to its greater tillering ability and below ground root competition.

The hop plant is also attacked by different pests and diseases, mainly Hop Mosaic Virus generated by aphids, Hop Damson Aphid (also known as *Phorodon humuli apterae*), Red Spider Mite, Verticillium wilt, caused by soil-borne fungi, and other fungal infections such as downy and powdery mildew.^32^

## 3 Use of Pesticides on Barley and Hop

Integrated pest management (IPM) uses a mixture of different practices to manage insects, pathogens, and weeds, so that the reliance on one control technique is reduced, ensuring this tool’s effectiveness for future use. A sequence of agronomic, biological, and chemical methods will usually be most effective and cost-efficient.

Pesticides (mainly insecticides and fungicides) are broadly used in different mixtures at many stages of crop growth and during postharvest storage.^33^ Internationally, there has been a significant change in attitude in the management of pests. The initial trend to select nonchemical strategies for crop protection seen at the end of the last century has turned into the official pest management policy in many countries. The negative environmental and human health impact of pesticides is currently being reduced through the application of different programs on pesticide management such as residue analysis, elimination of outdated stocks of pesticides, and means to dispose them and use of biopesticides among others.

However, the use of pesticides on barley and hop plants makes it possible to reach good yields, reducing losses during storage.^34^ Sulfonyl urea, pyrethroids, and triazoles among others are the most frequentl types of herbicides, insecticides, and fungicides, respectively, used on barley and hop. The problem is that residual levels of these pesticides in barley may persist in beer, although residues may also come from the soil itself and the water used because water is its major component (88%–92%).^35^

During the first step (malting), pesticide residues can remain on malt as pointed out by some authors.^36−38^ Subsequently, after the mashing and boiling steps, pesticides on the malt can pass into the wort in different amounts, depending on the process used, although it should be highlighted that the removal of trub and spent grains tends to decrease pesticide levels because most of them have low solubility in water.^39−44^ An excellent study can be found in the paper published by Inoue et al.^45^ where the fate of 368 pesticide residues was investigated during beer brewing (Table 1). Only a few pesticides remained at large ratios in beer. Specially, methamidophos with a high water solubility (200 g L^–1^) remained at about 80%, 2-(1-naphthyl)acetamide and imazaquin remained at 70%–80%, and fluoroxypyr, flumetsulam, thiamethoxam, imibenconazole-desbenzyl, imidacloprid, and tebuthiuron remained at 60%–70%. According to their physical properties, these nine pesticides (log *K*_OW_ < 2) largely remained in unhopped wort. Log *K*_OW_ (the logarithm base-10 of the partition coefficient between *n*-octanol and water) is commonly used in environmental fate studies as an indicator that a compound will bioaccumulate. Hence, special care should be taken with these nine pesticides and their use on raw materials, especially on malt destined for beer-making.

**Table 1:** Carryover of 368 Pesticide Residues during the Different Stages of Brewing^45^

number of pesticides				
carryover (%)	unhopped wort	spent grains	cooled wort	beer
0–10	186	23	241	261
11–30	47	26	41	51
31–50	27	27	20	27
51–80	29	112	21	16
>80	16	124	1	1
total	305	312	324	356
validation failure	63	56	44	12

Finally, if pesticide residues, particularly some fungicides, are dissolved in the brewer wort, some organoleptic alterations can be produced in the finished beer, provoking hazardous effects for the consumer.^46−53^

## 4 Analysis of Pesticide Residues in Raw Materials and Beer

Chemical analysis of food contaminants is a complex yet crucial endeavor, with wide-reaching implications in quality control, legislation, safety, and human health The large use of pesticides in cereals and hop has led the presence of pesticide residues in beer. Public apprehension over pesticide residues in malt beverages and beers has become a significant food safety issue. Regular analyses of the composition of the raw materials used in malting and brewing processes aim to assist quality control (QC).^54^ The main objective of quality management is to develop knowledge and understanding and identify suitable methods for the evaluation of product quality agreeing to the specifications of international quality standards.^55^ For this, multiresidue methods (MRMs) are required to identify and quantitate as many pesticides as possible in the most cost-effective manner.

**Figure 2:**
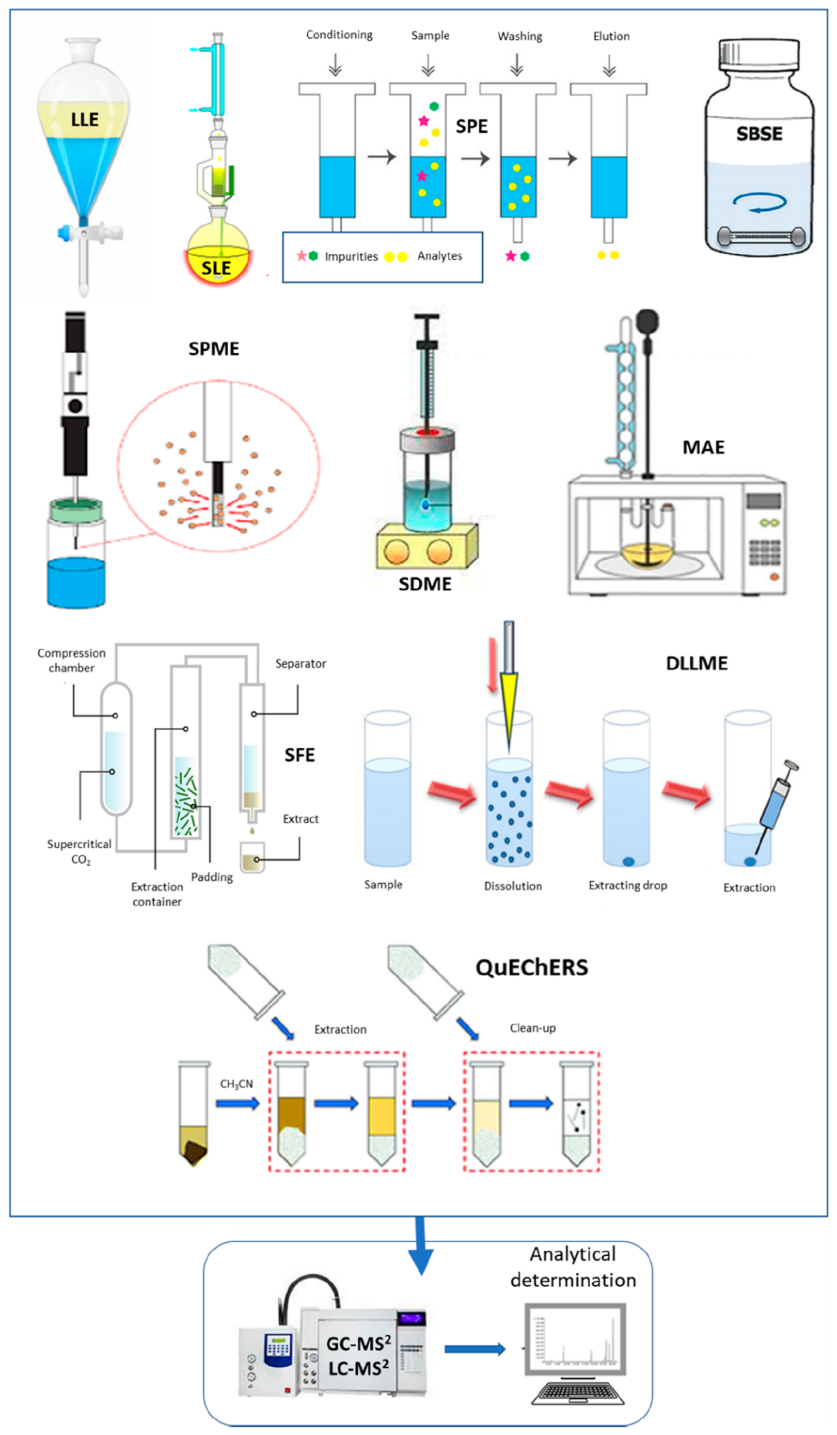
Summary of the main methods used for pesticide residue analysis.

Methods of sample preparation entail the following steps: sample collection, extraction techniques, and cleanup procedures. Isolation of pesticide residues from the matrix can be attained by different methods currently available in the scientific literature. Traditional liquid–liquid solvent extraction (LLE), solid–liquid extraction (SLE), or ultrasonic solvent extraction (USE) methods have been gradually substituted in the last years by more modern sample preparation methods. The evolution in extraction methods and improvement in the analytical techniques have reduced the complexity of the sample treatment, increasing the accuracy and precision of the analysis at the same time. The development of green analytical chemistry in addition to the concept of sustainable development led to a complete range of novel and alternative extraction procedures like solid phase extraction (SPE), matrix solid-phase dispersion (MSPD), solid-phase microextraction (SPME), dispersive solid-phase microextraction (DSPME), microwave assisted extraction (MAE), supercritical fluid extraction (SFE), single-drop microextraction (SDME), stir-bar sorptive extraction/twister (SBSE), dispersive liquid–liquid microextraction (DLLME) and/or QuEChERS (Quick, Easy, Cheap, Effective, Rugged, and Safe).^56^Figure 2 summarizes the main techniques used for pesticide residue analysis.

Chromatography techniques, mainly gas chromatography (GC) and liquid chromatography (LC), are usually used in the analysis of pesticide residues in different matrixes due to their high selectivity, separation efficiency, and good resolution. Most commonly residues in extracts are separated by GC using different detectors such as, nitrogen phosphorus (NPD), electron capture (ECD), flame ionization (FID), flame photometric (FPD), electrolytic conductivity (ELCD) or atomic emission (AED), and LC using detectors such as ultraviolet (UVD) dyode-array (DAD), fluorescence (FLD), or electrolytic conductivity (ELCD). However, although these element-selective detectors (ESDs) provide low detection limits (μg L^–1^ or μg kg^–1^) and are relatively easy to manage, the obtained data do not offer sufficient information to confirm the presence of a particular pesticide with certainty and reliability. For this reason and considering the universal nature of mass spectrometric detection (MSD), a mass spectrometer offers supporting information and increases reliability in the compound identity. For this purpose, different ionization sources such as electron impact (EI), chemical ionization (CI), atmospheric-pressure chemical ionization (APCI), atmospheric pressure photoionization (APPI), or electrospray ionization (ESI) coupled to different analyzers such as quadrupole (Q), ion trap (IT), triple quadrupole (TQ/QqQ), time-of-flight (TOF), and/or quadrupole-time-of-flight (Q-TOF) are commonly used.^57^ Even with selected ion monitoring (SIM), where multiple ions are monitored (MIM), the matrix may contain similar ions at the same retention time, so more rigorous selectivity must be raised to remove the matrix ions from the mass spectrum, which eliminates false positives and raises concentration values from matrix interferences. MS/MS does just that by ejecting all but the ion of interest out of the group. Then, a collision-induced dissociation (CID) energy is applied to fragment the ion into a unique product ion spectrum.^58^ However, although many established MRMs for the analysis of food samples have used GC, some water-soluble pesticides that may be very important in beverages, like wine or beer, are not appropriate for analysis or their recoveries are very low. For these water-soluble pesticides, there are many analytical methods using other chromatographic techniques such as high-performance liquid chromatography (HPLC), supercritical fluid chromatography (SFC), ultrahigh-performance liquid chromatography (UHPLC), or ultra-performance convergence chromatography (UPC^2^). A lot of analytical methods using this technique have been proposed in recent years to analyze pesticide residues in cereals, malt, hop, and beer.^44,45,47,59−67^

## 5 Evolution of Pesticide Residues during Brewing

Figure 1 summarizes the main steps of beer-making. More detailed information is described by Eaton.^68^ Depending upon the chemical nature of the residue in the RAC and the type of process involved, differences in the nature of the residue in the processed commodities and the RAC may be determined. Once the parent compounds and relevant metabolites have been identified, processing studies are conducted with RACs that normally undergo processing in the home or under industrial conditions. The process may be only physical or may involve the use of heat or chemicals.^19^ These types of processing are proposed to (i) generate evidence on the transfer of residues from RACs to the processed product, in order to estimate reduction (concentration) factors, (ii) supply a more realistic estimate to be made of the dietary intake of pesticide residues, and (iii) establish maximum residue limits (MRLs) in processed foodstuffs if necessary.

### 5.1 Dissipation of Pesticide Residues during Storage of Barley, Malt, and Spent Grains

If proper application methods of pesticides are not respected, their residues on barley can be above the MRL (maximum concentration of a pesticide residue, expressed as mg kg^–1^, to be legally permitted in or on food commodities and animal feeds based on Good Agricultural Practices) set by legislation. To protect the health of the consumers and facilitate world trade, the Food and Agriculture Organization (FAO) and the World Health Organization (WHO) of the United Nations (UN) have set a joint FAO/WHO Codex Alimentarius Commission (CAC) to coordinate food standards and establish universal MRLs.^69^ Desmarchellier et al.^70^ described the losses of several pesticides (bioresmethrin, carbaryl, fenitrothion, *d*-fenothrin, methacrifos, and pirimiphos-methyl) from barley after storage and malting finding losses ranging from 58% to 100%. Other authors pointed that, after insecticide (phentoate, fenitrothion, and ethiofencarb) and fungicide (triflumizole, mepromil, propiconazole, and triadimefon) application on field barley, more than 80% of phentoate and fenitrothion (organophosphorus insecticides) residues persisted after 2 months of grain storage at room temperature while the loss of other pesticides varied from 28% to 85%, increasing slightly the amount of metabolites of triadimefon (triadimenol) and triflumizole (TF-6-1).^36^ Different models^71−73^ are commonly used to explain pesticide decay in different matrixes. Possibly, the most used model to describe pesticide losses on grain protectants is the single first order (SFO) model, according to the following equation:
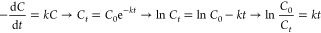
where *t* is the reaction time, *C*_0_ the initial concentration of the pesticide, *C*_*t*_ is its residual concentration at time *t*, and *k* is the rate constant.^74,75^

Navarro et al.^37^ observed a great linear correlation (*r* > 0.95) between ln *C*_*t*_ and *t* when they studied the dissipation of several pesticides over 3 months of malt storage at 20 ± 2 °C. Moreover, an excellent correlation (*r* > 0.99) between analytical and theoretical concentration calculated (*C*_0_) at *t*_0_ was noted, which suggests that the SFO model is appropriate. Based on the calculated values for *k*, the following dissipation rate was observed: myclobutanil > propiconazole > fenitrothion > trifluralin > pendimethalin > malathion > nuarimol with half-lives fluctuating from 244 to 1533 days.

To study the disappearance rate of seven pesticides in the spent grains, Navarro et al.^41,42^ evaluated their decay during storage (3 months). In all cases, a great linear correlation (*r* > 0.96) between residue level and time was found. The necessary times to reach their respective MRLs in barley ranged from 408 to 958 days for nuarimol and propiconazole (fungicides), respectively, showing a high persistence for all pesticides except for malathion (insecticide), whose residual level was below of the corresponding MRL.

### 5.2 Fate of Pesticide Residues during Malting

The malting process involves three basic stages: (i) steeping, (ii) germination, and (iii) kilning.^68,76^

Table 2 shows bibliographical data extracted from the scientific literature relating to pesticide decay during malting. Although, generally, steeping significantly decreased pesticide residues, the carryovers for pendimethalin and trifluralin (dinitroaniline herbicides) varied from 80% to 85% into steeped grains. Both herbicides are hydrophobic compounds (log *K*_OW_ > 5) with low water solubility (0.2–0.3 mg L^–1^). Consequently, a small proportion of their residues (10%–15%) were removed during steeping. Concerning to the organophosphorus pesticides, 55% of malathion (log *K*_OW_ = 2.7) was eliminated from barley grains after steeping while 48% of fenitrothion residues (log *K*_OW_ = 3.5) was removed in this stage.^37^ Other studies show a carryover of 43% for fenitrothion after steeping step, while other organophosphorus insecticide as phentoate remains in lower proportion (27%), as pointed out by Miyake et al.^36^ Contrarily, other organophosphorus insecticides as pirimiphos-methyl persist in high proportion (90%) after steeping.^77^ Some fungicides such as nuarimol (pyrimidine), myclobutanil, and propiconazole (triazole) were removed from the barley grains (after steeping) in percentages varying from 30% to 41%, which it is expected according to their respective *K*_OW_ values,^37^ while Miyake et al.^36^ noticed higher percentages of removal (50%–76%) for some azole fungicides such as propiconazole, triflumizole, triadimenol, and triadimefon.

**Table 2:** Remaining Amounts (%) of Some Pesticide Residues during Malting

		stage			
pesticides	log *K*_OW_^a^	steeping	germination	kilning	references
cyproconazole	3.1	47	38	31	(38)
diniconazole	4.3	70	61	39	(38)
epoxiconazole	3.4	62	53	38	(38)
ethiofencarb	2.0	3	1	5	(36)
fenitrothion	3.4	52	31	13	(37)
flutriafol	2.3	43	35	30	(38)
malathion	2.8	45	20	14	(37)
mepronil	3.8	24	6	30	(36)
myclobutanil	2.9	59	42	36	(37)
nuarimol	3.2	64	57	51	(37)
pendimethalin	5.2	85	67	49	(37)
phentoate	3.7	27	4	18	(36)
propiconazole	3.6	50	10	55	(36,37)
		55	43	30	
tebuconazole	3.7	56	45	37	(38)
triadimefon	3.1	24	5	30	(36)
triadimenol	3.1	36	13	47	(36)
triflumizole	4.4	38	11	9	(36)
trifluralin	5.3	80	65	50	(37)

a See ref (99).

**Figure 3:**
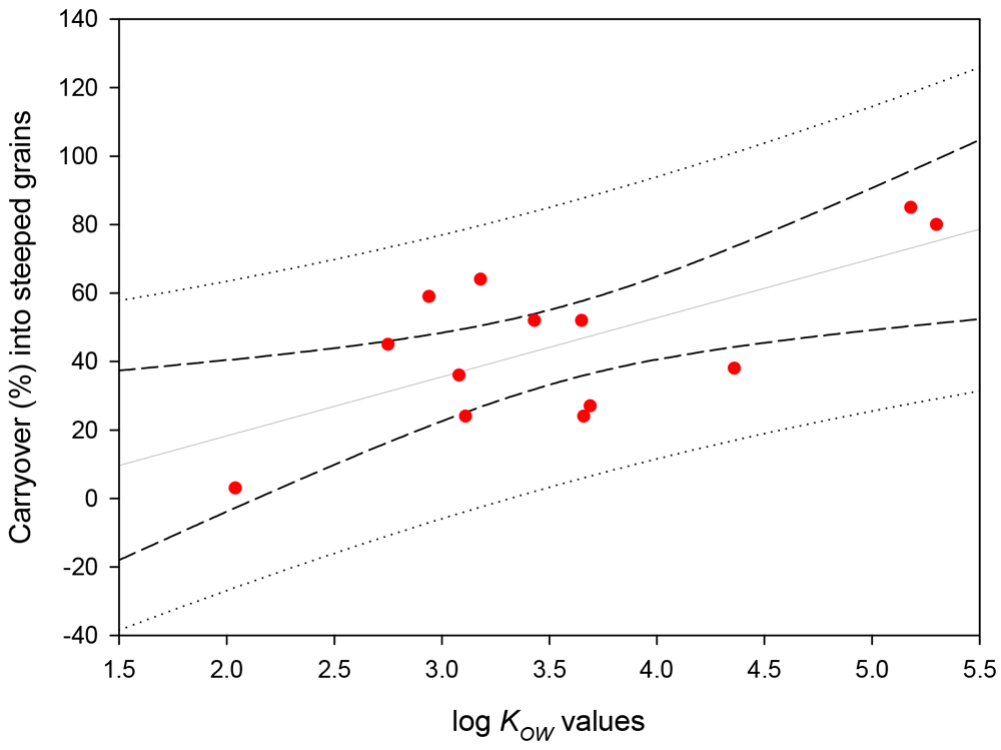
Correlation between remaining amounts (%) of some pesticides after steeping and their log *K*_OW_ values according to the data shown in Table 2 (short dash line is 95% confidence interval and dotted line 95% prediction interval).^35^

These data are supported by the correlation between amounts removed after steeping and log *K*_OW_ of the pesticides, as can be seen in Figure 3. Other sterol biosynthesis-inhibiting (SBI) fungicides such as cyproconazole, diniconazole, epoxiconazole, flutriafol, and tebuconazole were removed after steeping, ranging from 30% to 57%.^38^ The more hydrophilic fungicides (flutriafol and cyproconazole) were removed at the end of this step in higher quantities than the more hydrophobic compounds (diniconazole, tebuconazole, and epoxiconazole). The calculated transfer factor (TF, the ratio of the residue concentration in the processed commodity to that in the raw agricultural commodity) for cyproconazole and flutriafol was estimated to be 0.4, while for the other triazole fungicides it was near 0.5. The carryover of hydrophilic pesticides (low log *K*_OW_) such as malathion was lower, while carryovers of hydrophobic pesticides (pendimethalin and trifluralin) were higher. Miyake et al.^40^ recommend that brewers should pay particular attention to the residues of hydrophilic pesticides on malt with *K*_OW_ values below 4 because they can be carried over into beer being of special interest the steeping stage of malting. The same authors^40^ showed that pesticides with log *K*_OW_ > 2 can persist on malt. Therefore, the control of pesticides with log *K*_OW_ values ranging from 2 to 4 is crucial for maltsters and brewers.

### 5.3 Removal of Pesticide Residues during Mashing

Generally, about 200 g of grain is needed to obtain 1 L of wort at 12° Plato, although this amount fluctuates depending on the desired alcoholic content. Therefore, residues existing in the grain, even if fully transferred to the beer, should undergo dilution by a factor of 5, although the log *K*_OW_ values of pesticides should be considered.^40^ Since the low water solubility of most pesticides and their tendency to be easily adsorbed on the suspended matter, as it happens during wine making, residues in beer are expected to be very low.^13^

The remaining residues for some pesticides after mashing process are shown in Table 3. Soluble substances (amino acids, peptides, and sugars) formed during malting and mashing steps are extracted into the sweet wort (liquid fraction), which is then separated from the spent grains (residual solid particles). Agreeing with Navarro et al.,^41^ at the end of the mashing stage, the remaining percentages of three fungicides (myclobutanil, nuarimol, and propiconazole) were below 10% of the amount verified in malt, with propiconazole showing the greatest reduction (to 4%). Contrarily, the residual amounts on spent grain were comparatively high (38%, 42%, and 26% for myclobutanil, propiconazole, and nuarimol, respectively, all the compounds having log *K*_OW_ > 2). Comparable behavior was noted for atrazine and terbuthylazine during mashing, when 55% and 80%, respectively, were retained on spent grains.^39^

**Table 3:** Remaining Amounts (%) of Some Pesticide Residues after Mashing and Boiling^a^

pesticide	log *K*_OW_^b^	sweet wort	spent grains	brewer wort	spent hops	references
atrazine	2.5	45	55	42	20	(39)
α-BHC	4.0	8	54	30	15	(40)
captafol	3.8	BDL^c^	3	BDL	BDL	(40)
chlorpyrifos	4.7	17	3	4	32	(40)
cyproconazole	3.1	10	40	9	ND^d^	(41)
deltamethrin	4.6	BDL	45	3	37	(40)
dichlorvos	1.9	8	BDL	BDL	BDL	(40)
diclofuanid	3.7	10	10	BDL	BDL	(40)
dicofol	4.3	BDL	70	18	60	(40)
diniconazole	4.3	4	49	3	ND	(41)
epoxiconazole	3.4	8	44	7	ND	(41)
fenitrothion	3.4	4	30	3	ND	(42)
fenobucarb	2.8	35	30	64	1	(40)
fenvalerate	5.0	BDL	50	3	7	(40)
flucythrinate	6.2	BDL	60	BDL	10	(40)
flutriafol	2.3	13	36	10	ND	(38)
glyphosate	–3.2	97	3	95	2	(40)
malathion	2.7	20	35	15	5	(40,42)
		7	40	4	ND	
myclobutanil	2.9	9	38	8	ND	(41)
nuarimol	3.2	6	26	6	ND	(41)
oxamyl	0.4	1	BDL	20	BDL	(40)
parathion-methyl	3.0	1	BDL	10	3	(40)
pemdimethalin	5.2	1	21	1	ND	(42)
permethrin	6.1	BDL	70	BDL	50	(40)
pirimicarb	1.7	84	14	50	3	(40)
pirimiphos-methyl	4.2	2	68	6	12	(40)
propiconazole	3.6	4	42	4	ND	(41)
tebuconazole	3.7	8	44	7	ND	(38)
terbutylazine	3.2	12	80	7	40	(39)
triadimenol	3.1	36	ND	ND	ND	(78)
trifluralin	5.3	1	17	1	ND	(42)

a More information can be consulted in the paper by Inoue et al.^45^ where the fate of 368 pesticide residues was investigated during beer brewing.

b See ref (99).

c Below detection limit.

d Not determined.

**Figure 4:**
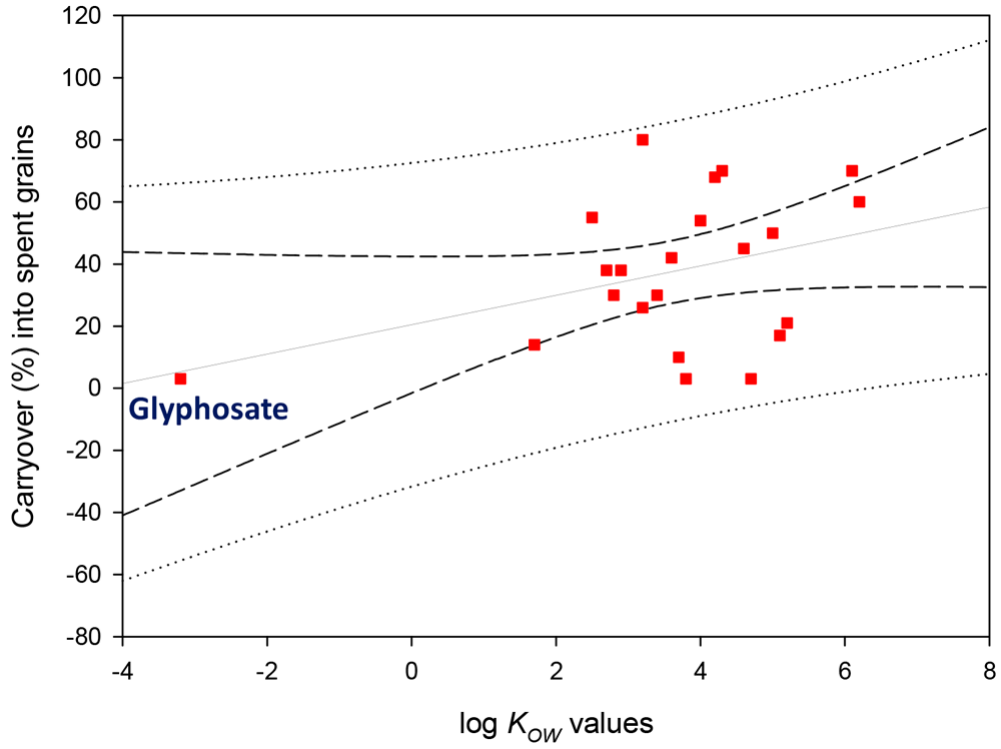
Correlation between remaining amounts (%) of some pesticides after mashing and their log *K*_OW_ values according to the data shown in Table 3 (short dash line is 95% confidence interval and dotted line 95% prediction interval).^35^

Figure 4 shows the correlation between the carryover of some pesticides in spent grains and their respective log *K*_OW_. As general rule, adsorption affinity is directly related to their polarities: the more polar the pesticide, the lower the adsorbed amount observed.^47^ It is important to point out that malt and adjuncts maceration generates a high amount of suspended matter, which could adsorb residues and, if the detected levels allow it, the spent grains are commonly used as animal feeds, which it implies a commercial practice for this byproduct. As shown in Table 3, the residual amounts of dinitroaniline herbicides nearly disappear after mashing (<1% of the initial amount in sweet wort), while the remaining percentages of organophosphorus insecticides, fenitrothion and malathion, in sweet wort were greater, at 4% and 7%, respectively.^42^ Contrarily, the recovered amounts on spent grain were quite high (21%, 17%, 30%, and 40% for pendimethalin, trifluralin, fenitrothion, and malathion, respectively). Other water-soluble pesticides such as glyphosate (organophosphorus) and pirimicarb (carbamate) were found in sweet wort in amounts higher than 80%, while no residues of pyrethroid compounds (fenvalerate, deltamethrin, permethrin, and flucythrinate) were detected in sweet wort because they were retained on the spent grains. For oxamyl, dichlorvos, parathion-methyl, chlorpyrifos, dichlofuanid, and captafol noticeable losses were observed, possibly due to evaporation, thermal degradation, and/or chemical reactions with some wort components during mashing.^40^ Azole fungicides (cyproconazole, diniconazole, epoxiconazole, flutriafol, myclobutanil, propiconazole, tebuconazole, and triadimenol) were recovered from the sweet wort in proportions varying from 3% to 36% for diniconazole and triadimenol, respectively while about 40%–50% of the initial mass on malt was found in spent grains.^38,41,78^ The calculated TFs after mashing indicated the strong dissipation of triazole fungicide residues from malt to sweet wort (TFs ≤ 0.02). As previously specified, a greater amount of residues was retained on the spent grains. Consequently, TFs for spent grains were noticeably high (0.66–0.89 for flutriafol and diniconazole, respectively).^38^ It is important to highlight that the spent grains, a moist byproduct from the brewing industry, is ideal for blending with other forage supplies to simulate dry matter and an excellent feed for cattle and sheep.^79^

### 5.4 Decrease of Pesticide Residues during Boiling

Table 3 shows the carryovers for different pesticides in brewer wort and spent hops. As can be observed, a minor reduction (<10%) was detected in the residual content after boiling for myclobutanil, nuarimol, and propiconazole, which reveals the stability of the three pesticides at high temperature (>100 °C).^41^ Similar conduct was observed for other azole fungicides.^38^ The remaining amounts of pendimethalin and trifluralin detected in the brewer wort were lower than 30% of their content in sweet wort after wort boiling. Regarding the fall of organophosphorus pesticides, the residual levels of fenitrothion and malathion were 83% and 65% of the content in wort after mashing.^42^ Other authors^40^ have pointed that the percentages of residues for fenobucarb, glyphosate, and pirimicarb into the cold wort were remarkably high, showing a great stability at temperatures over 100 °C, while dicofol and pyrethroid insecticides were mostly recovered from the spent hops and dichlorvos, dichlofuanid, and captafol totally disappear, probably due to their low stability at the temperature reached during the boiling step. On the other hand, the totality of oxamyl, parathion-methyl, and chlorpyrifos residues in cold wort and spent hops was slightly higher than that of the mashing process. Authors supposed that this behavior in the basis of these pesticides can react with some components in the sweet wort but not in the cold wort.

The fate of pesticide residues from hop to wort during boiling has also been considered. Some authors have confirmed that pesticides added to hop were not detected in the young beer after wort boiling.^40^ Another study carried out by Navarro et al.^61^ shows that fenamiphos, malathion, and methidathion residues were below their detection limits in the young beer after the addition of enriched hop pellets (2 μg g^–1^) to the wort boiling, while 1 μg L^–1^ was recovered for fenarimol. In the study carried out by Walsh et al.,^80^ of the pesticides (boscalid, dimethomorph, bifenazate, pyraclostrobin, triflumizole, quinoxyfen, and etoxazole) noticed on conventionally treated hops, only two pesticides (boscalid and bifenazate) were detected in the beers at above the level of quantification that could be statistically analyzed, and these amounts were orders of magnitude below levels with any health or legal ramifications. In most situations, the nonexistence of pesticide residues is owing to their losses during boiling and the high dilution of hop. Other work carried out with field-treated hop including different pesticides show that residues of tebuconazole and Z- and E-dimethomorph were lower than 31% of the predictable amount, bearing in mind that was only the diluted residues. Successive analysis demonstrated that 84%–109% of quinoxyfen, chlofenapyr, pyridaben, tebuconazole, fenarimol, and both Z- and E-dimethomorph continue on the spent hops,^47^ which is explained by the presence of a high amount of lipophilic components in hop, mainly resins and waxes. In EU, processing studies on hop are only mandatory when residual levels are higher than 5 mg kg^–1^ in dried cones because the high dilution factor (over 250).^19^

**Figure 5:**
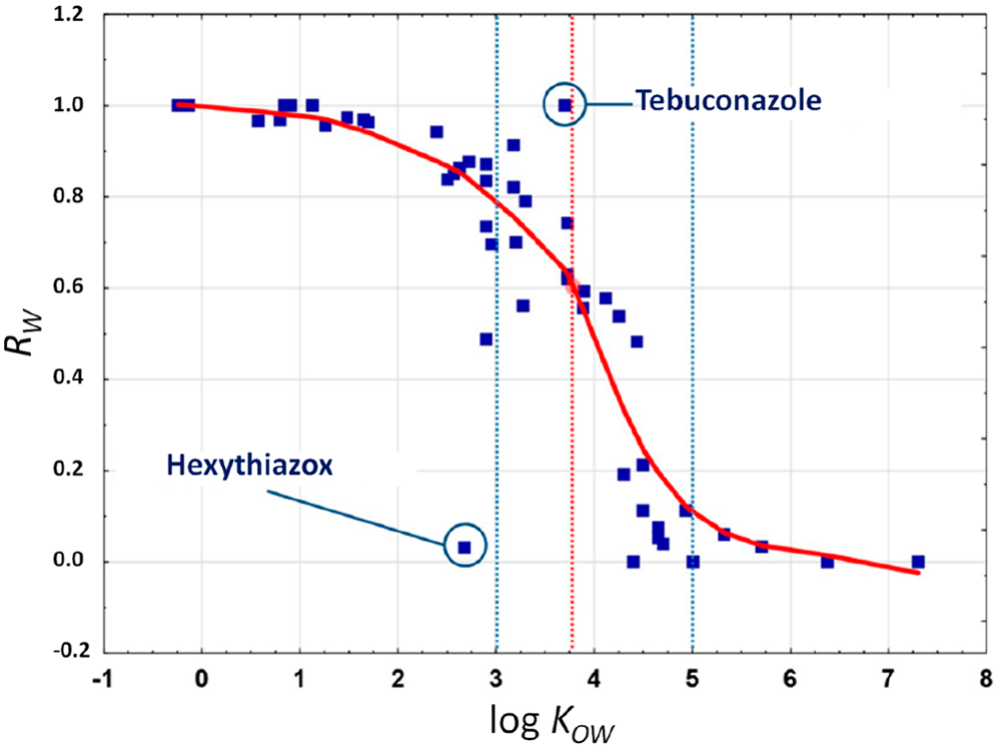
LOWESS correlation (red line) between the log *K*_OW_ value and the measured residual ratio (*R*_W_) in wort showing marked inflection points of the correlation curve.^44^

The correlation between the log *K*_OW_ values and the residual ratios (*R*_W_) of 58 pesticides associated with hops to estimate their carryover into brewed beer was assessed by Dušek et al.^44^ to forecast their behavior during wort boiling. *R*_W_ was considered on the basis of pesticide amount in hopped wort related to the sum of amounts of the pesticide in spent hops and hopped wort. Figure 5 show the correlation between *R*_W_ and log *K*_OW_ values for all pesticides included in groups A (pesticide carryovers into hopped wort the amount spiked on hop were ≥ 50%) and B (pesticides remained in spent hop or were extracted from <50%), excluding those pesticides not detected (below detection limit). The relationship between these values was evaluated using LOWESS (locally weighted scatterplot smoothing) regression analysis and is depicted by a smooth curve through the data points.

The results show that water-soluble pesticides (log *K*_OW_ < 3) were isolated at >70%, while pesticides with log *K*_OW_ < 2 were almost entirely extracted from hop to wort. The point of inflection of LOWESS regression was at log *K*_OW_ = 3.75 equiv to extraction efficacy of 60%. Accordingly, the pesticides with log *K*_OW_ < 3.75 were most likely extracted, and contrariwise, the pesticides with log *K*_OW_ > 3.75 most likely remained on hop. The extraction efficiencies of hexythiazox (3%) and tebuconazole (100%) showed that water solubility (0.5 and 36 mg L^–1^, respectively) of these pesticides had a greater impact that their log *K*_OW_ values (2.7 and 3.7, respectively). Therefore, the log *K*_OW_ values could be a valuable tool for prediction of the extraction efficacies and would be consistent for pesticides with low log *K*_OW_ (<3) because larger log *K*_OW_ values indicate diminishing extraction efficiency, which is more affected by other physical properties of the pesticides as previously pointed by different authors during malting and mashing.^36,37,45,78^

### 5.5 Decline of Pesticide Residues during Primary Fermentation

Regarding the impact of the alcoholic fermentation on the removal of pesticide residues (Table 4), a considerable decrease was observed for propiconazole residues (48% of the amount recovered in brewer wort) but much less for other fungicides such as nuarimol and myclobutanil (over 20%).^41^ On the other hand, no residues of dinitroaniline herbicides were detected in young beer fermented with bottom-yeasts, while there was a notorious decrease in the cases of organophosphorus insecticides, fenitrothion and malathion (65% and 42% of the content recorded in brewer wort, respectively).^42^ For Miyake et al.,^40^ no significant decrease was observed during the fermentation process for some groups of pesticides. Other pesticides such as captafol, chlofenapyr, deltametrin, dicofol, fenvalerate, flucytrinate, permetrin, pyridaben, and quinoxyfen show a strong decay after addition to the pitching wort, being below detection limit at the end of fermentation, while tebuconazole, fenarimol, and both Z- and E-dimethomorph had relatively high recoveries, varying their carryovers at the end of fermentation from 41% to 75%.^47^ Similarly, some triazole fungicides had relatively high residue recoveries (53%–82%) once fermentation was complete using top-fermenting yeasts.^81^ Flutriafol (log *K*_OW_ = 2.3) and cyproconazole (log *K*_OW_ = 3.1) persisted in the beer after fermentation, in proportions about 80%. On the contrary, tebuconazole, epoxiconazole, and diniconazole (log *K*_OW_ = 3.4–4.3) were removed from the beer in higher proportions, mainly associated with trub. Other experiments showed that top-fermenting yeasts (*Saccharomyces cerevisiae*) had a superior capacity to convert triazine herbicide residues into their hydroxylated metabolites to bottom-fermenting yeasts (*Saccharomyces carlsbergensis)*.^39^

**Table 4:** Carryover (%) of Some Pesticide Residues after Fermentation^a^

pesticide	log *K*_OW_^b^	young beer	spent yeast	references
atrazine	2.5	95^c^, 76^d^	ND^e^	(39)
α-BHC	4.0	110^c^	30	(40)
captafol	3.8	BDL^f^	BDL	(40)
chlorfenapyr	4.8	BDL	34	(47)
chlorpyrifos	4.7	12^c^	16	(40)
cyproconazole	3.1	79^c^	ND	(81)
deltamethrin	4.6	BDL	15	(40)
dichlorvos	1.9	65^c^	BDL	(40)
diclofuanid	3.7	17^c^		(40)
dicofol	4.3	BDL	10	(40)
*E*-dimethomorph	2.6	75^c^	23	(47)
*Z*-dimethomorph	2.7	70^c^	22	(47)
diniconazole	4.3	53^d^	ND	(81)
epoxiconazole	3.4	59^d^	ND	(81)
fenarimol	3.7	41^c^	48	(41)
fenitrothion	3.4	35^c^	ND	(42)
fenobucarb	2.8	94^c^	BDL	(40)
fenvalerate	5.0	BDL	11	(40)
flucythrinate	6.2	BDL	2	(40)
flutriafol	2.3	82^d^	ND	(81)
glyphosate	–3.2	110^c^	BDL	(40)
malathion	2.7	58^c^	2	(42)
		20^c^	ND	
myclobutanil	2.9	78^c^	ND	(41)
nuarimol	3.2	82^c^	ND	(41)
oxamyl	0.4	30^c^	BDL	(40)
parathion-methyl	3.0	60^c^	4	(40)
pemdimethalin	5.2	BDL	ND	(42)
permethrin	6.1	BDL	11	(40)
pirimicarb	1.7	50^c^	BDL	(40)
pirimiphos-methyl	4.2	40^c^	6	(40)
propiconazole	3.6	52^c^	ND	(41)
pyridaben	6.4	BDL	43	(47)
quinoxyfen	4.7	BDL	62	(47)
tebuconazole	3.7	55^c^	58	(47)
		67^d^		(81)
terbutylazine	3.2	100^c^	ND	(39)
		50^d^		
triadimenol	3.1	43^c^	ND	(78)
trifluralin	5.3	BDL	ND	(42)

a More information can be consulted in the paper by Inoue et al.^45^ where the fate of 368 pesticide residues was investigated during beer brewing.

b See ref (99)

c Values obtained using bottom-yeasts.

d Values obtained using top-yeasts.

e Not determined.

f Below detection limit.

**Figure 6:**
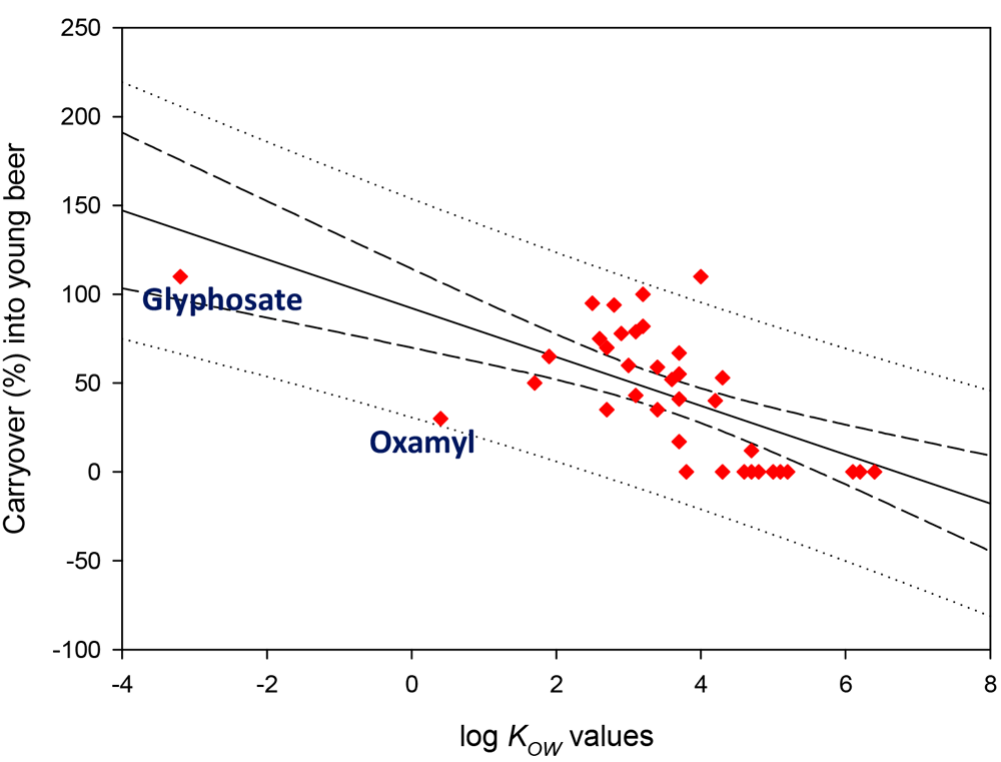
Correlation between remaining amounts (%) of some pesticides after fermentation and their log *K*_OW_ values according to the data shown in Table 4 (short dash line is 95% confidence interval and dotted line 95% prediction interval).^35^

These results suggest a correlation between log *K*_OW_ and the number of pesticides found in the young beer and the trub, as can be seen in Figure 6. It is remarkable the high carryover of glyphosate (about 100%) due to their hydrophilic properties (water solubility >100 g L^–1^ and log *K*_OW_ < −3).^82^ Glyphosate has been catalogued as carcinogen (Group 2A) by IARC, and it has been found in beer.^83^ The effect of the yeast (biotic metabolism) and the anaerobic environment created by fermentation (abiotic degradation) are responsible of the losses during fermentation.^84^ In addition, agreeing to the Henry’s Law constants (*H*, the predisposition of a compound to volatilize from aqueous solution to air), those pesticides with low water solubility and high vapor pressure may escape to the atmosphere.^85^ This effect is also favored by the constant increment of CO_2_ during the first days of fermentation.

**Figure 7:**
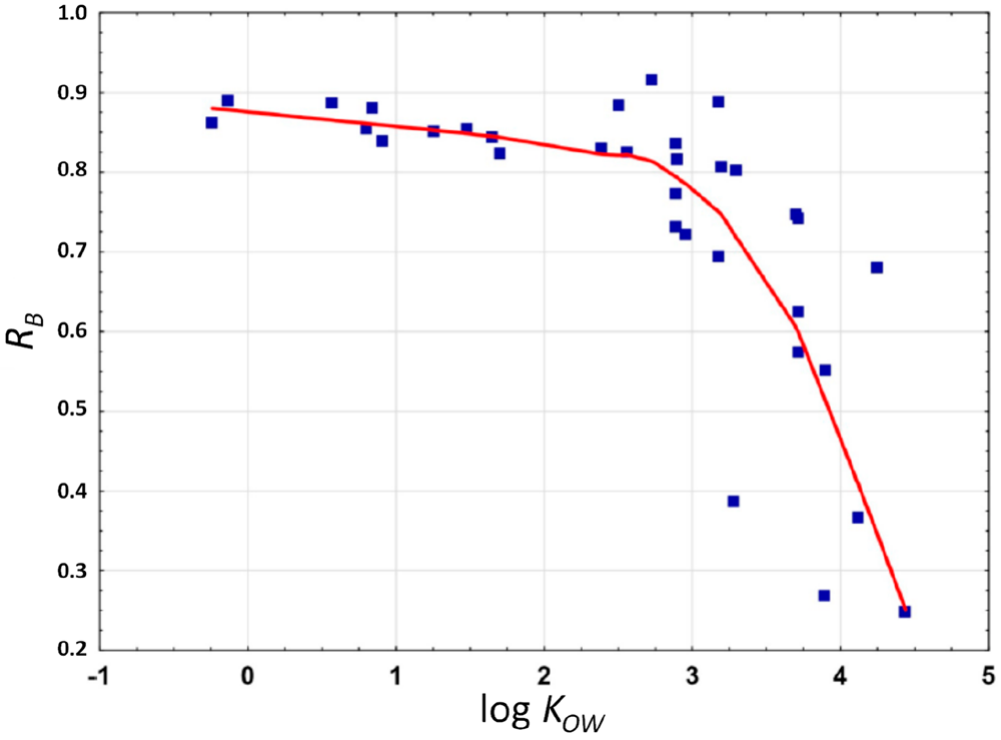
LOWESS correlation (red line) between the log *K*_OW_ value and the measured residual ratio (*R*_B_) in beer.^44^

In the brewing trial conducted by Dušek et al.^44^ with pesticide spiked hop, the concentration of their residues was assessed in hopped wort preceding the addition of yeast and after 7 days and 4 weeks of fermentation. All 33 pesticides carried over into hopped wort were detected in young beer remaining at various rates of initial to final concentration, which appeared to be likewise related to their log *K*_OW_ values. The LOWESS correlation between log *K*_OW_ and *R*_B_ (calculated as pesticide amount in beer related to the sum of pesticide amount in hopped wort and beer) depicted in Figure 7 shows that pesticides with a log *K*_OW_ < 3 tend to persist in the final beer at approximately 80%. However, pesticide residues with elevated log *K*_OW_ values fell during fermentation, up to about 25% of initial concentration, and showed short correlation with their log *K*_OW_ values. Diflubenzuron (log *K*_OW_ = 3.89) remained at 27% and fludioxonil (log *K*_OW_ = 4.12) remained at 37%, in contrast to tebufenozide (log *K*_OW_ = 4.25) that persisted in beer at 68%. The decrease in the pesticide levels with log *K*_OW_ > 3 can be explained by their adsorption on yeast cells during the first stage of fermentation and/or by their aqueous hydrolysis at pH (<5) of beer occurring during the secondary fermentation (6 weeks).

**Figure 8:**
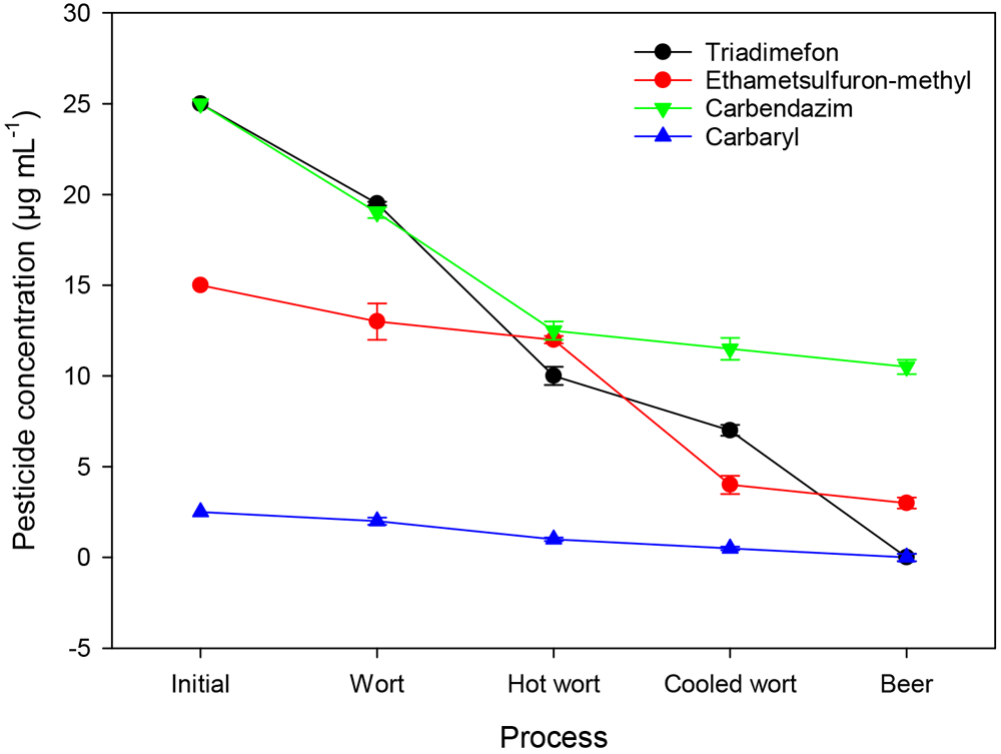
Changes in concentration of different pesticide residues during beer brewing.^53^

In a study carried out by Wei et al.,^53^ among different four types of pesticides (Figure 8), triadimefon and carbendazim (25 μg mL^–1^) reduced significantly by 22% and 23%, respectively, during the saccharification process, barely affecting the brewing process. This is because they were retained into spent grains during the separation of wort and spent grain. However, only 13% of ethametsulfuron-methyl and 10% of carbaryl (15 and 2.5 μg mL^–1^, respectively) were removed in the saccharification process, showing slightly inhibition on saccharification and considerably negative impacts on yeast growth and alcohol fermentation. Wort boiling could take away the pesticides largely except ethametsulfuron-methyl (7%). Triadimefon, carbendazim, and carbaryl were reduced by 38%, 28%, and 35%, respectively, because heat treatment in saccharification and boiling process could cause adsorption, volatilization, pyrolysis, or hydrolysis of pesticides.^45^ After filtration, the wort and spent grain were separated and most of the pesticides remained in the spent grain. Consequently, the pesticide residues were mostly reduced before fermentation. After fermentation, triadimefon and carbaryl residues were practically absent. However, the concentration of ethametsulfuron-methyl and carbendazim after fermentation persisted (3.5 and 9.7 μg mL^–1^, respectively). This may be attributed to the good chemical stability of both.

### 5.6 Evolution of Pesticide Residues during Lagering (maturation phase), Filtration, and Beer Aging

No significant reduction on the residual levels has been observed in any case after maturation and filtration. Nuarimol reduced its concentration (by 10%) regarding the young beer.^41^ On the other hand, fenitrothion and malathion decreased their contents regarding the young beer by 33% and 37%.^42^ Hack et al.^39^ neither found loss of triazine herbicides after filtration.

As for other foods, also for beer, different quality attributes may be subject to changes during storage. Unlike some wines, beer aging is frequently judged negative for flavor quality.^86^ After 3 months of storage, the concentrations of some pesticides like propiconazole and fenitrothion fell strongly (50% and 75%, respectively), while the reduction observed for myclobutanil and nuarimol was less pronounced (<25% of the initial amount in young beer) and malathion residues were their below detection limit.^41,42^

## 6 Effect of Pesticide Residues on the Beer Quality

Some fermentation byproducts have a significant impact on the flavor, aroma, taste, color, and other organoleptic properties of the beer. Some micropollutants, such as pesticides, can modify the ordinary fermentative process, being able to originate in some cases sluggish and even stuck fermentation. Consequently, the organoleptic properties of the beer should be altered as occurs in other fermented beverages as wine.^87,88^

Flavor appraisal is a very important control point in the quality control of beer. There are two types of sensory analysis: (i) subjective (by means of human senses) and (ii) objective (instrumental analysis), which are commonly used to assess the organoleptic properties of beer. Drink quality mainly depends on its sensory characteristics, which are evaluated by human sensory preferences.^89^ The sensory analysis by a panel of well-trained tasters is one of the most significant tools. In some cases, the harsh astringent flavor detected in some beer samples is due to some metabolites derived from the parent pesticides present in the raw materials. Hence, residues of up to 5 mg kg^–1^ of carbaryl were found on treated barley and up to 41 μg L^–1^ of carbaryl-derived 1-naphtol were recovered from beer. Elimination of up to 90% of the carbaryl and 1-naphtol occurred during malting. Some tasters were able to reliably distinguish beer containing 20 μg L^–1^ of 1-naphtol.^46^

**Figure 9:**
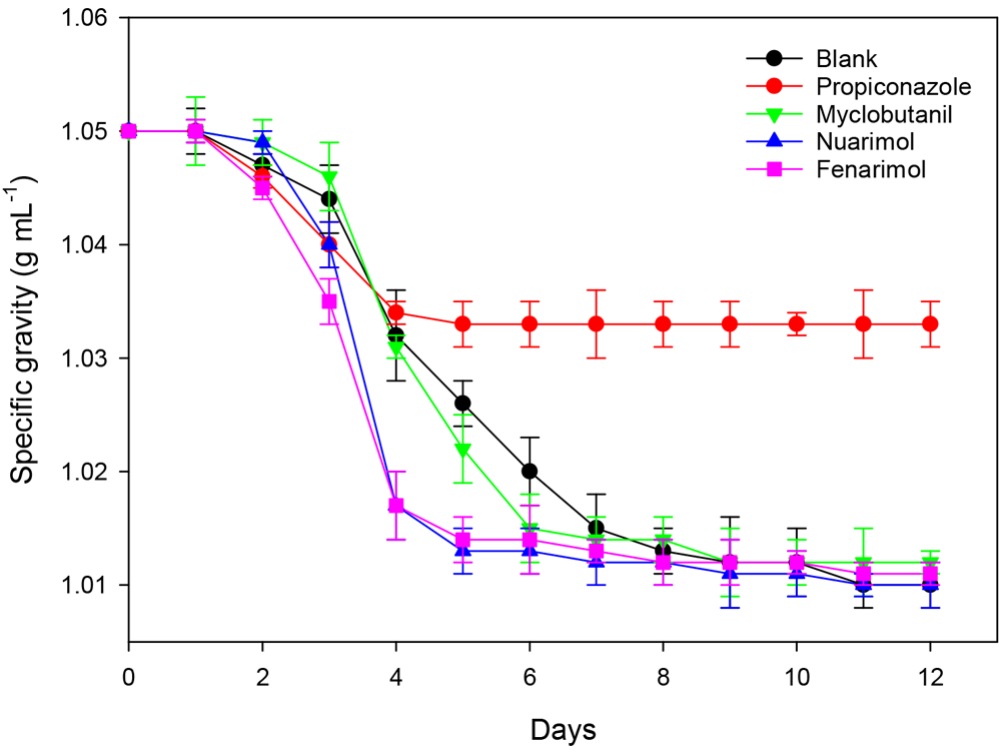
Evolution of specific gravity (*n* = 3) vs time during lager fermentation for blank and samples treated with pyrimidine and triazole fungicides.^48^

According to Navarro et al.,^48^ a noticeable impact of certain pesticides in the fermentation rate (lager fermentation) has been observed as depicted in Figure 9, where the progression of specific gravity with time is shown for both blank and treated samples. As can be observed, from the fourth day onward, the fermentation precipitately ends (stuck fermentation, i.e., the premature finish of fermentation before all fermentable sugars have been metabolized) in the samples containing propiconazole residues as compared with the blank. However, no substantial differences in the evolution of specific gravity were found for samples fermented in the presence of myclobutanil residues, while in those containing nuarimol and fenarimol residues, the fermentative kinetic was quicker from days 2 to 6, possibly owing to the rapid assimilation of nitrogen by the yeasts.

**Figure 10:**
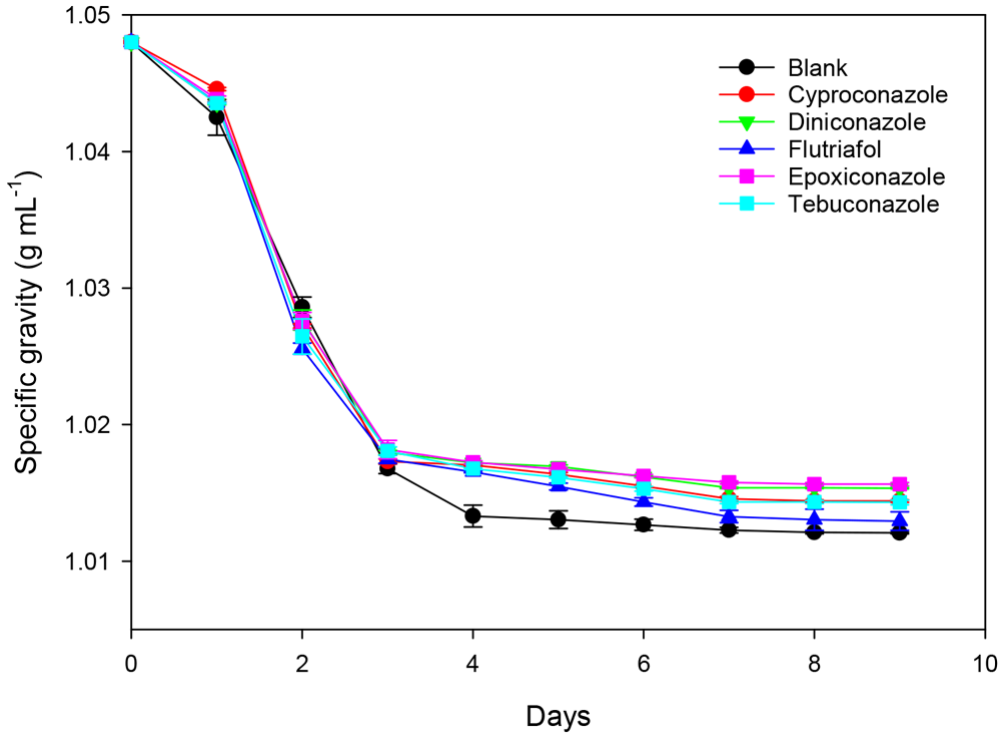
Evolution of specific gravity (*n* = 3) vs time during ale fermentation for blank and samples treated with triazole fungicides.^81^

Other results (Figure 10) show that, at the end of ale fermentation, the mean values of specific gravity for the blank samples were significantly different (*p < 0.05*) from those measured in the samples containing residues of triazole fungicides.^81^ Some authors have suggested that the complex nitrogen composition of the medium may generate similar conditions to those liable for inducing sluggish/stuck fermentation.^90^

**Figure 11:**
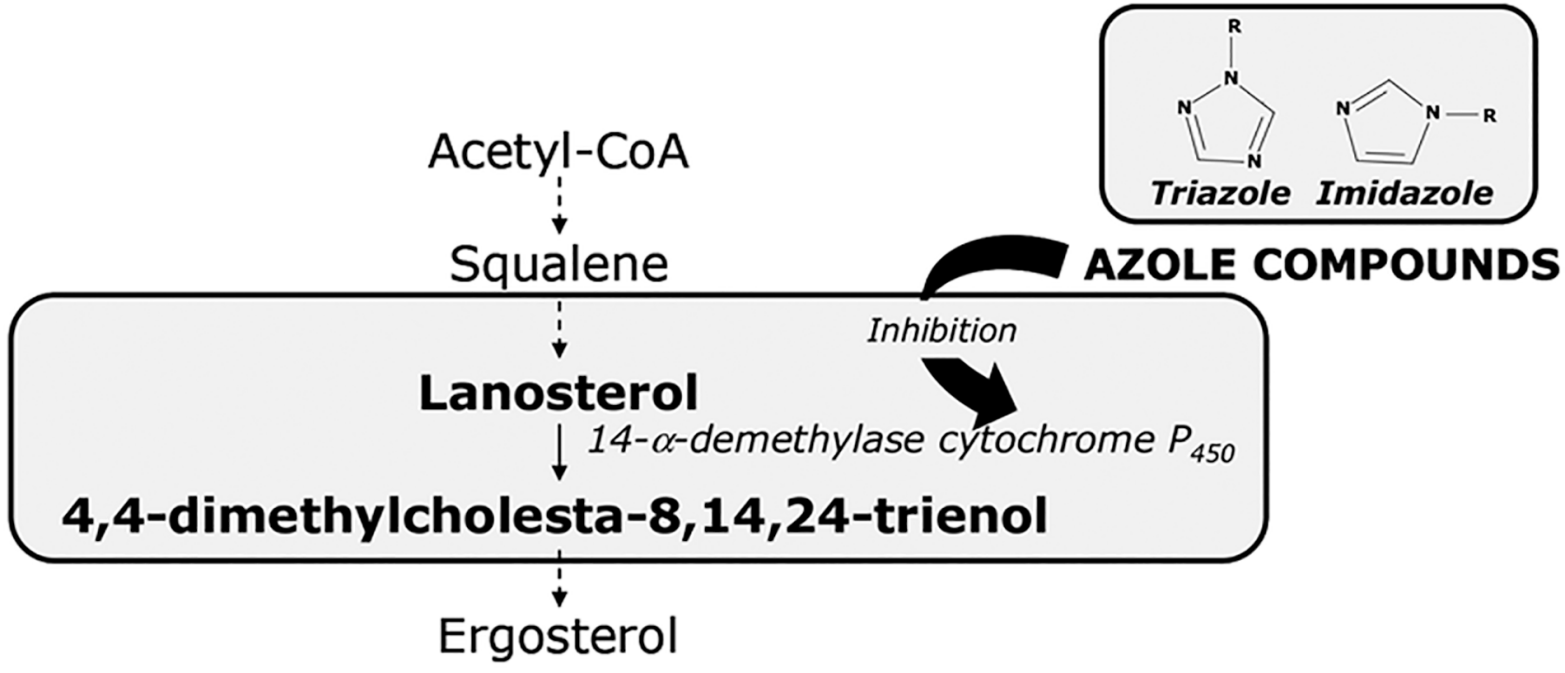
Schematic inhibition of the ergosterol biosynthesis by azole compounds.^51^

Concretely, triazole- and imidazole-derivatives (azole compounds) have a leading role as antifungals agents in agriculture because of their low toxicity and broad therapeutic spectrum. Triazole-derivatives used for agricultural purposes are effectively used against mildews and rust of cereal grains, vegetables, ornamentals, and fruits. They are SBIs, and its antifungal action is centered on their aptitude to interfere with steroid biosynthesis and thereby with the formation of fungal walls.^91^ They act by inhibiting the cytochrome P450 (CYP51, lanosterol C_14_ α-demethylase) mediated conversion of lanosterol to ergosterol, a crystalline sterol synthesized by yeast from sugars, resulting in accumulation of sterols still bearing α-C_14_ methyl group, altering the exact shape and physical properties of the fungal membrane and producing permeability changes and failure of membrane imbedded proteins.^92^Figure 11 shows the schematic inhibition of the ergosterol biosynthetic pathway.

**Figure 12:**
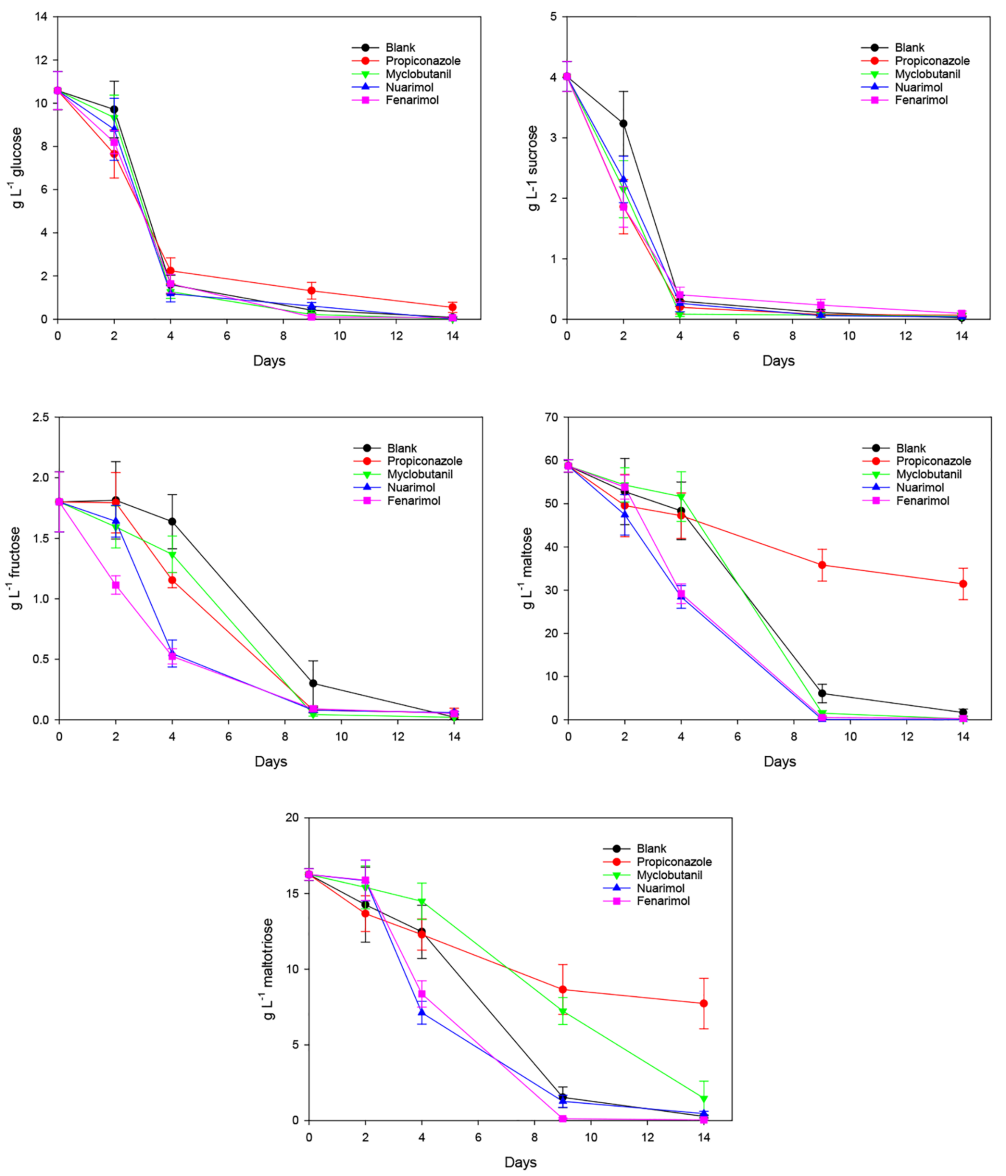
Changes in the content of fermentable carbohydrates (*n* = 3) vs time during lager fermentation for blank and samples treated with pyrimidine and triazole fungicides.^48^

Not all sugars in wort are fermented in the same way and percentage. Figure 12 shows the progress of fermentable carbohydrates during fermentation, agreeing with Navarro et al.^48^ Since yeasts must hydrolyze sugar polymers before it can use them, they always attack hexoses first. Thus, the yeasts assimilate a great amount of glucose during the first 96 h. No significant differences (*p* < 0.05) were detected between the blank sample and those with nuarimol, fenarimol, and myclobutanil residues, while whether in the case of propiconazole assay where a delay in the glucose consumption was detected after 4 days. Sucrose was easily metabolized by yeasts in all cases because the enzyme responsible for its breakdown (invertase) is located in the cell wall and sucrose is, therefore, consumed as a start of fermentation sugar by the yeast. No significant differences (*p* < 0.05) were observed between the blank and the other samples although assimilation of this sugar was something slower in the blank sample during the first 48 h. Fructose assimilation follows a different pattern to glucose and sucrose. Samples with fenarimol and nuarimol (pyrimidine fungicides) residues consume this sugar faster that those containing triazole fungicide (myclobutanil and propiconazole) residues. The slowest assimilation agrees to the blank sample. In all cases, the higher consumption occurs from 24 to 216 h. Comparable behavior exhibits maltose although the greatest consumption takes place between 96 and 216 h, during the main fermentation. It is important to comment that, after the fourth day, the consumption by the yeasts of this sugar was drastically reduced in the sample with propiconazole residues, which is expected because fermentation was paused at this time. Finally, maltotriose was the last sugar assimilated by the yeasts. No significant differences (*p* < 0.05) were detected when evaluating the behavior of the blank sample and those containing residues of fenarimol and nuarimol. On the other hand, triazole fungicides, especially propiconazole, had a prominent influence on the assimilation of this sugar by the yeasts. Also, a higher amount of residual sugars (glucose, fructose, maltose, and maltotriose) was found in the beer obtained in the presence of residues of dinitroaniline herbicides (pendimethalin and trifluralin) and organophosphorus insecticides (fenitrothion, malathion, and methidathion).^49^ Similar findings were found during the fermentation of young ale beer in the presence of triazole fungicide (cyproconazole, diniconazole, epoxiconazole, flutriafol, and tebuconazole) residues where a higher content of residual sugars (essentially maltose and maltotriose) was recovered in the beer.^81^

The influence of some pesticide residues on the pH and color of the beer has also been studied by Navarro et al.^48^ Hence, the pH values at the end of the fermentation were 4.1 (blank sample) and 3.0, 3.7, 3.8, and 3.9 for beer including residues of propiconazole, myclobutanil, fenarimol, and nuarimol. Also in this case, the presence of propiconazole sensibly modifies the beer quality. Similarly, significant differences (*p* < 0.05) were observed for pH and color of the beer after fermentation among blank and samples containing residues of organophosphorus insecticides.^49^ pH values below 4.0 originate an acidic beer taste, mainly caused by microbial infections during fermentation. As a result of a decrease in pH during fermentation, several colloidally dissolved bitter substances and polyphenols can precipitate on the CO_2_ bubbles in the foam head or as a result of adsorption on the yeast cells.^27^ As a consequence of their low solubility at a pH below 5 and temperatures lower than 10 °C, the α-acids not isomerized during the boiling of the wort precipitate. In this way, as pointed out by Navarro et al.,^48^ the values of bitterness were below its detection limit in all cases. The same authors have pointed out that the color of the beer falls about 1–1.5 EBC units during lager fermentation. This is probably due to the discoloration of some substances due to the fall in pH and absorption of highly colored compounds in the yeast cells or precipitation in the container bottom.^27^ For ale fermentation samples containing triazole fungicides, the color intensity was lower and tint higher than the values in blank samples.^81^

Regarding the flavonoid and total polyphenol contents detected after fermentation, significant differences (*p* < 0.05) were observed between the samples containing residues of triazole fungicides and the others, especially in the case of propiconazole due to the stuck fermentation caused after 4 days of the beginning. However, no significant differences (*p* < 0.05) were detected for the pH and polyphenol content after fermentation of young ale beer (top-fermenting yeasts) among the blank and the treated samples with five triazole fungicides.^81^ It is important to remark that several papers investigate the association between chemical content of wine and beer and beneficial health effects for the consumer. Thus, Piazzon et al.^93^ reported on the phenolic acid content in different types of beer and assigned the antioxidant power of bock, abbey, and ale beers to the higher content of polyphenols and phenolic acids. In other cases, the metabolization of carbaryl to 1-naphtol during brewing confers a characteristic harsh astringent flavor to the beer.^46^

According to the above mentioned, if the pitching wort contains SBIs, especially triazole compounds, it is important to use fining agents such as bentonite, activated charcoal, or polyvinylpirrolidone (PVPP) to remove or at least reduce their amount in the wort since they can alter the beer quality. Some results pointed out by Pérez et al.^94^ demonstrate that the use of activated charcoal reduces significantly the level of pesticides in the wort. In fact, more than 80% of myclobutanil and 70% of propiconazole residues were removed.

## 7 Toxicological Risk of Pesticide Residues on Beer

Human exposure to synthetic chemicals like pesticides is a growing issue in the developed world, worsened through industrialization. Sometimes, the presence of some metabolites generated during the brewing stages have the same or even more toxicity than their parent pesticides, and they can persist during fermentation. Pesticide metabolites are ordinarily water-soluble because most of them have amine or hydroxyl groups.^95,96^ This is the case for triadimenol and TF-6-1, metabolites of triadimefon and triflumizole, respectively, both found in beer.^78^ Particularly 1*H*-1,2,4-triazole (TA), a common metabolite of some triazole fungicides, is a compound with high water solubility (700 g L^–1^) and stability (pH = 5–9, 25 °C) for more than 30 days, and it is known to cause a problem on reproduction and development.^82^ A similar behavior was observed for triazine herbicides such as atrazine and terbuthylazine, where hydroxy analogues (OHA and OHT) were predominantly detected in top-fermented beers. Checking of these herbicides, mainly in the water used for brewing is critical because, like atrazine, these polar degradation products are catalogued as possible human carcinogens.^39^

The ethylene bisdithiocarbamate (EBDC) or propylene bisdithiocarbamate (PBDC) fungicides are often used to exemplify the generation of toxicologically relevant metabolites during food processing. The conversion of EBDCs and PBDCs to ethylenethiourea (ETU) and propylenethiourea (PTU) is mostly favored by high pH and heat,^19^ although the formation of ETU by thermal degradation in aqueous medium can be greatly reduced by the addition of copper sulfate due to the formation of a stable cupric ethylene bisdithiocarbamate complex.^97^ Research carried out with hop treated with radiolabeled EBDCs showed that parent fungicides (maneb/propineb) were mainly degraded to ETU/PTU, both showing carcinogenic effects.^98^ Consequently, studies to know the behavior of pesticide residues during brewing are necessary to perform a more realistic dietary risk assessment.

Bearing in mind the above-mentioned, we can affirm that the cultivation of barley and hop is negatively affected by bacteria fungus, virus, and pests. For this reason, many pesticides, mainly insecticides and fungicides, are extensively used in different mixtures at many stages of growing and during postharvest storage. Consequently, the monitoring and surveillance of pesticide residues during brewing is an emerging issue for human and animal health. Beer is one of the-most common drink worldwide (in 2020, the global beer consumption was 177.5 million kL with a decrease of about 12.8 million kL due to effects from the spread of COVID-19). Furthermore, with the more data generated in the brewing process during the last years, the more accurately beer types could be created to ideally meet the taste expectations of consumers in certain occasions. This all has revolutionized the development process in breweries, and new processing techniques are being used. In addition, some byproducts of the brewing industry like spent grains are mostly used as animal feed. For these reasons, studies on the behavior and fate of pesticide residues during beer-making are very useful to safeguard the health of consumers and animals.

Although processing steps have the ability to introduce or produce new pollutants, the contrary may also be true because certain contaminants present in raw materials may be degraded or eliminated. Most of the pesticides used on barley and hop reduce their residual concentrations after brewing and are not identified in the finished product (beer). Only a small content of those pesticides with log *K*_OW_ < 3 having hydrophilic properties have the possibility of remaining in the unhopped (sweet) wort. Such a decrease in the residual level is mainly due to their adsorption onto spent grain. On the other hand, the thermal stability and percentage of dissipation of pesticides show different decay rates. The amount of the parent compounds measured in samples during boiling of sweet wort serves as a basis of the extent of their thermal stability being able to be categorized from stable to nonstable based on the percentage removed between initial and final concentrations determined approximately after 2 h of boiling. The losses during fermentation stage may be attributed to the yeast (biotic metabolism) and anaerobic environment created by fermentation (abiotic degradation). Some pesticide residues can alter the usual fermentative process being able to cause, in certain cases, sluggish and even stuck fermentation and consequently modifying some organoleptic properties such as residual sugar content, pH, color, bitterness, or polyphenol content among others. No significant reduction on the residual levels of pesticides has been observed after maturation and filtration.

The pesticides in some groups can show the same behavior, whereas those of different other classes did not. For example, pesticides belonging to the benzoylurea and/or pyrethroid groups are largely adsorbed onto spent grain due to their hydrophobic properties (high log *K*_OW_ values). On the other hand, the compounds included in the neonicotinoid group, which are hydrophilic (low log *K*_OW_ values), barely adsorbed onto spent grain and remain even in fermented beer. However, although pesticides of the sulfonylurea group are hydrophilic, they disappear completely after the wort is boiled, indicating that they are decomposed by temperature. Unlike these groups, pesticides such as carbamates, organophosphates or triazoles have different log *K*_OW_ values and chemical stabilities even within the same group. Therefore, they did not show similar behavior during brewing. Consequently, a theoretical risk management based only on chemical class is not conclusive. Finally, it is crucial to monitor the generation of toxicologically relevant metabolites derived from the parent compounds to avoid consumer health risks.

## Abbreviations Used

AED: atomic emission detector
APCI: atmospheric-pressure chemical ionization
APPI: atmospheric-pressure photoionization
CAC: Codex Alimentarius Commission
CI: chemical ionization
CID: collision-induced dissociation
COVID: Coronavirus disease
DAD: dyode-array detector
DMG: Danish Malting Group
DSPME: dispersive solid-phase microextraction
EBC: European Brevery Convention
EBDC: ethylene bisdithiocarbamate
EC: European Commission
ECD: electron capture detector
EFSA: European Food Safety Authority
EI: electron impact
ELCD: electrolytic conductivity detector
ESDs: element selective detectors
ESI: electrospray ionization
ETU: ethylenethiourea
EU: European Union
FAO: Food Agricultural Organization
FID: flame ionization detector
FLD: fluorescence detector
FPD: flame photometric detector
GC: gas chromatography
GLP: good laboratory practice
GMP: good manufacturing practice
HPLC: high-performance liquid chromatography
IARC: International Agency for Research on Cancer
IPM: integrated pest management
LC: liquid chromatography
LLE: liquid–liquid extraction
LOWESS: locally weighted scatterplot smoothing
MAE: microwave assisted extraction
MIM: multiple ion monitoring
MRLs: maximum residue limits
MRMs: multiresidue methods
MS: mass spectrometry
MSD: mass spectrometric detector
MSPD: matrix solid-phase dispersion
NPD: nitrogen–phosphorus detector
OHA: hydroxy analogues
OHT: hydroxy triazol
OTA: ochratoxin A
PBDC: propylene bisdithiocarbamate
PPP: plant protection product
PTU: propylenethiourea
PVP: polyvinylpirrolidone
Q: quadrupole
QC: quality control
Q-TOF: quadrupole-time of flight
QuEChERS: quick, easy, cheap, effective, rugged, and safe
RAC: raw agricultural commodity
SBI: sterol biosynthesis inhibitor
SBSE: stir-bar sorptive extraction
SFC: supercritical fluid chromatography
SFO: single first order
SIM: selected ion monitoring
SLE: solid–liquid extraction
SPE: solid phase extraction
SPME: solid-phase microextraction
TA: 1*H*-1,2,4-triazole
TF: transfer factor
TMDI: theoretical maximum daily intake
TOF: time of flight
TQ/QqQ: triple quadrupole
UHPLC: ultrahigh-performance liquid chromatography
UN: United Nations
UPC^2^: ultra-performance convergence chromatography
USE: ultrasonic solvent extraction
UVD: ultraviolet detector
WHO: World Health Organization

## References

[ref1] Donatelli M.; Magarey R. D.; Bregaglio S.; Willocquet L.; Whish J. P. M.; Savary S. Modelling the impacts of pests and diseases on agricultural systems. Agric Syst. 2017, 155, 213–224. 10.1016/j.agsy.2017.01.019. 28701814 PMC5485649

[ref2] Akashe M. M.; Pawade U. V.; Nikam A. V. Classification of pesticides: a review. Int. J. Res. Ayurveda Pharm. 2018, 9 (4), 144–150. 10.7897/2277-4343.094131.

[ref3] Tudi M.; Daniel Ruan H.; Wang L.; Lyu J.; Sadler R.; Connell D.; et al. Agriculture development, pesticide application and its impact on the environment. Int. J. Environ. Res. Public Health. 2021, 18 (3), 1112 10.3390/ijerph18031112. 33513796 PMC7908628

[ref4] Popp J.; Petö K.; Nagy J. Pesticide productivity and food security. A review. Agron Sustain Dev. 2013, 33 (1), 243–255. 10.1007/s13593-012-0105-x.

[ref5] Sabarwal A.; Kumar K.; Singh R. P. Hazardous effects of chemical pesticides on human health-Cancer and other associated disorders. Environ. Toxicol Pharmacol. 2018, 63, 103–114. 10.1016/j.etap.2018.08.018. 30199797

[ref6] Nguyen T. T.; Roselló C.; Bélanger R.; Ratti C. Fate of residual pesticides in fruit and vegetable waste (FVW) processing. Foods. 2020, 9 (10), 1468 10.3390/foods9101468. 33076324 PMC7602544

[ref7] Food and Agriculture Organization (FAO) of the United Nations. International Code of Conduct on the Distribution and Use of Pesticides. https://www.fao.org/3/y4544e/y4544e.pdf (accessed 2022-07-18).

[ref8] Abubakar Y; Tijjani H; Egbuna C; Adetunji C. O.; Kala S; Kryeziu T. I., Pesticides, history, and classification. In Natural Remedies for Pest, Disease and Weed Control; Egbuna C, Sawicka B, Eds.; Academic Press: Cambridge, MA, 2020. 10.1016/B978-0-12-819304-4.00003-8

[ref9] EC. Regulation (EC) No 1107/2009 of the European Parliament and of the Council of 21 October 2009 concerning the placing of plant protection products on the market and repealing Council Directives 79/117/EEC and 91/414/EEC.; OJEU, 2009;Vol. L 309, pp 1–50.

[ref10] Comparison of cumulative dietary exposure to pesticide residues for the reference periods 2014–2016 and 2016–2018. EFSA J. 2021, 19 (2), e06394 10.2903/j.efsa.2021.6394. 33598048 PMC7869021

[ref11] Boobis A. R.; Ossendorp B. C.; Banasiak U.; Hamey P. Y.; Sebestyen I.; Moretto A. Cumulative risk assessment of pesticide residues in food. Toxicol. Lett. 2008, 180 (2), 137–150. 10.1016/j.toxlet.2008.06.004. 18585444

[ref12] Leong W.-H.; Teh S.-Y.; Hossain M. M.; Nadarajaw T.; Zabidi-Hussin Z.; Chin S.-Y.; Lai K.-S.; Lim S.-H. E. Application, monitoring and adverse effects in pesticide use: The importance of reinforcement of Good Agricultural Practices (GAPs). J. Environ. Manage. 2020, 260, 109987 10.1016/j.jenvman.2019.109987. 32090796

[ref13] Farris G. A.; Cabras P.; Spanedda L. Pesticide residues in food processing. Ital J. Food Sci. 1992, 4 (3), 149–169.

[ref14] Holland P. T.; Hamilton D.; Ohlin B.; Skidmore M. W. Effects of storage and processing on pesticide residues in plant products. Pure Appl. Chem. 1994, 66 (2), 335–356. 10.1351/pac199466020335.

[ref15] Kaushik G.; Satya S.; Naik S. N. Food processing a tool to pesticide residue dissipation-A review. Food Res. Int. 2009, 42 (1), 26–40. 10.1016/j.foodres.2008.09.009.

[ref16] Keikotlhaile B. M.; Spanoghe P.; Steurbaut W. Effects of food processing on pesticide residues in fruits and vegetables: a meta-analysis approach. Food Chem. Toxicol. 2010, 48 (1), 1–6. 10.1016/j.fct.2009.10.031. 19879312

[ref17] González-Rodríguez R. M.; Rial-Otero R.; Cancho-Grande B.; Gonzalez-Barreiro C.; Simal-Gándara J. A. Review on the fate of pesticides during the processes within the food-production chain. Crit. Rev. Food Sci. Nutr. 2011, 51 (2), 99–114. 10.1080/10408390903432625. 21328107

[ref18] Bajwa U.; Sandhu K. S. Effect of handling and processing on pesticide residues in food. A review. J. Food Sci. Technol. 2014, 51 (2), 201–220. 10.1007/s13197-011-0499-5. PMC3907644 24493878

[ref19] Timme G; Waltz-Tylla B. Effects of food preparation and processing on pesticide residues in commodities of plant origin. In Pesticide Residues in Food and Drinking Water: Human Exposure and Risks; Hamilton D, Crossley S, Eds.; John Wiley & Sons, Ltd.: West Sussex, UK, 2003; pp 121–148. 10.1002/0470091614.ch1

[ref20] Mandelbaum D. G. Alcohol and culture. Curr. Anthropol. 1965, 6 (3), 281–293.

[ref21] Stewart G. G. Beer: Raw materials and wort production. In Encyclopedia of Food and Health; Caballero B, Finglas P. M., Toldrá F, Eds.; Academic Press: Cambridge (MA), 2016. 10.1016/B978-0-12-384947-2.00058-1

[ref22] Posada J. Cerveza y salud: investigaciones científicas que analizan el valor nutritivo de la cerveza y sus beneficios sobre la salud; Centro de Información Cerveza y Salud: Spain, 1998.

[ref23] Sendra J. M.; Carbonell J. V. Evaluación de las propiedades nutritivas, funcionales y sanitarias de la cerveza, en comparación con otras bebidas; Centro de Información Cerveza y Salud: Madrid, Spain, 1999.

[ref24] Baxter E. D.; Hughes P. S. Beer: Quality, Safety and Nutritional Aspects; Royal Society of Chemistry: Cambridge, UK, 2001.

[ref25] Fagrell B.; de Faire U.; Bondy S.; Criqui M.; Gaziano M.; Jackson R.; Klatsky A.; Salonen J.; Shaper A. G. The effects of light to moderate drinking on cardiovascular diseases. J. Int. Med. 1999, 246 (4), 331–340. 10.1046/j.1365-2796.1999.00576.x. 10583704

[ref26] Palmer G. H. Barley and Malt. In Handbook of Brewing, 2nd ed.; Graham G. S., Fergus G. P., Eds.; CRC Press: Boca Raton, FL, 2006; pp 139–160. 10.1201/9781420015171

[ref27] Kunze W. Technology of Brewing and Malting, 4th International ed.; VLB Berlin: Berlin, Germany, 2010.

[ref28] Fincher G. B.; Stone B. A. Physiology and Byochemistry of Germination in Barley. In Barley: Chemistry and technolog.; MacGregor A. W., Bhatty S. R., Eds.; American Association of Cereal Chemists, Inc.: St. Paul, MN, 1993; pp 247–295.

[ref29] Domínguez F. Plagas y enfermedades de las plantas cultivadas, 9th ed.; Mundi-Prensa: Spain, 2004.

[ref30] International Agency for Research on Cancer (IARC). Ochratoxin A. Some Naturally occurring substances: Food items and constituents, heterocyclic aromatic amines and mycotoxins. In Monographs on the Evaluation of Carcinogenic Risks to Humans; lARC Working Group on the Evaluation of Carcinogenic Risks to Humans: Lyon, France, 1993; Vol. 56, pp 489–521.

[ref31] Danish Malting Group (DMG). Malting Process. https://ens.dk/sites/ens.dk/files/Globalcooperation/dmgfaktaark.pdf (accessed 2022-08-22).

[ref32] Walsh D. B.; Gent D. H.; Barbour J. D.; Boyston R. A.; George A. E.; James D. G.; Sirrine J. R. Field guide for integrated pest management in hop, 3rd ed.; Hop Industry Plant Protection Committee: Pullman, WA, 2015.

[ref33] Theaker P. D.; Clarke B. J.; Currie B. R.; Gough A. J. Pesticides and brewing materials: reasons, regulations and residues. Tech Q Master Brew Assoc Am. 1989, 26 (4), 152–160.

[ref34] Biendl M. Systematic monitoring of residues. Brauwelt Int. 2017, 4, 257–260.

[ref35] Navarro S; Vela N. Fate of pesticide residues during brewing. In Beer in Health and Disease Prevention; Preedy V, Eds.; Academic Press, Elsevier, Inc.: San Diego, CA, 2009; pp 415–428. 10.1016/B978-0-12-373891-2.00040-7

[ref36] Miyake Y.; Hashimoto K.; Matsuki H.; Ono M.; Tajima R. Fate of insecticide and fungicide residues on barley during storage and malting. J. Am. Soc. Brew Chem. 2002, 60 (3), 110–115. 10.1094/ASBCJ-60-0110.

[ref37] Navarro S.; Pérez G.; Navarro G.; Vela N. Decline of pesticide residues from barley to malt. Food Add Contam. 2007, 24 (8), 851–859. 10.1080/02652030701245189. 17613072

[ref38] Navarro S.; Vela N.; Navarro G. Fate of triazole fungicide residues during malting, mashing and boiling stages of beermaking. Food Chem. 2011, 124 (1), 278–284. 10.1016/j.foodchem.2010.06.033.

[ref39] Hack M.; Nitz S.; Parlar H. Behavior of [^14^C] atrazine, [^14^C] terbuthylazine, and their major metabolites in the brewing process. J. Agric. Food Chem. 1997, 45 (4), 1375–1380. 10.1021/jf9605411.

[ref40] Miyake Y.; Koji K.; Matsuki H.; Tajima R.; Ono M.; Mine T. Fate of agrochemical residues, associated with malt and hop, during brewing. J. Am. Soc. Brew Chem. 1999, 57 (2), 46–54. 10.1094/ASBCJ-57-0046.

[ref41] Navarro S.; Pérez G.; Vela N.; Mena L.; Navarro G. Behavior of myclobutanil, propiconazole, and nuarimol residues during lager beer brewing. J. Agric. Food Chem. 2005, 53 (22), 8572–8579. 10.1021/jf0505832. 16248555

[ref42] Navarro S.; Pérez G.; Navarro G.; Mena L.; Vela N. Decay of dinitroaniline herbicides and organophosphorus insecticides during brewing of lager beer. J. Food Prot. 2006, 69 (7), 1699–1706. 10.4315/0362-028X-69.7.1699. 16865906

[ref43] Kong Z. Q.; Li M. M.; Chen J. Y.; Gui Y. J.; Bao Y. M.; Fan B.; Jian Q.; Francis F.; Dai X. Behavior of field-applied triadimefon, malathion, dichlorvos, and their main metabolites during barley storage and beer processing. Food Chem. 2016, 211, 679–686. 10.1016/j.foodchem.2016.05.058. 27283683

[ref44] Dušek M.; Jandovská V.; Olšovská J. Tracking, behavior and fate of 58 pesticides originated from hop during beer brewing. J. Agric. Food Chem. 2018, 66 (38), 10113–10121. 10.1021/acs.jafc.8b03416. 30175912

[ref45] Inoue T.; Nagatomi Y.; Suga K.; Uyama A.; Mochizuki N. Fate of pesticides during beer brewing. J. Agric. Food Chem. 2011, 59 (8), 3857–3868. 10.1021/jf104421q. 21401094

[ref46] Jones R. D.; Kavanagh T. E.; Clarke B. J. Determination of carbaryl residues in malt and beer and their impact on beer quality. J. Am. Soc. Brew Chem. 1988, 46 (2), 43–50. 10.1094/ASBCJ-46-0043.

[ref47] Hengel M. J.; Shibamoto T. Method development and fate determination of pesticide-treated hop and their subsequent usage in the production of beer. J. Agri Food Chem. 2002, 50 (12), 3412–3418. 10.1021/jf020089n. 12033804

[ref48] Navarro S.; Pérez G.; Navarro G.; Mena L.; Vela N. Influence of fungicide residues on the primary fermentation of young lager beer. J. Agric. Food Chem. 2007, 55 (4), 1295–1300. 10.1021/jf062769m. 17263547

[ref49] Navarro S.; Pérez G.; Navarro G.; Mena L.; Vela N. Variability in the fermentation rate and colour of young lager beer as influenced by insecticide and herbicide residues. Food Chem. 2007, 105 (4), 1495–1503. 10.1016/j.foodchem.2007.05.035.

[ref50] Navarro S.; Vela N.; Navarro G. Behaviour of pesticide residues from barley to beer: recent overview. Int. J. Food Process Technol. 2011, 1 (1), 1–16.

[ref51] Navarro S; Pérez-Lucas G; Vela N; Navarro G. Behavior of Triazole Fungicide Residues from Barley to Beer. In Processing and Impact on Active Components in Food; Preedy V, Ed.; Academic Press, Elsevier, Inc.: San Diego, CA, 2015; pp 525–532. 10.1016/B978-0-12-404699-3.00062-7

[ref52] Bartkiene E.; Juodeikiene G.; Zadeike D.; Baliukoniene V.; Bakutis B.; Cizeikiene D. Influence of microbial and chemical contaminants on the yield and quality of ethanol from wheat grains. J. Sci. Food Agric. 2019, 99 (5), 2348–2355. 10.1002/jsfa.9433. 30338535

[ref53] Wei Q.; Zhong B.; Zhu J.; Hu S.; He J.; Hong Q.; et al. Effect of pesticide residues on simulated beer brewing and its inhibition elimination by pesticide-degrading enzyme. J. Biosci Bioeng. 2020, 130 (5), 496–502. 10.1016/j.jbiosc.2020.07.003. 32758402

[ref54] Sherma J. Pesticide Residue Analysis: 1997–1998. J. AOAC Int. 1999, 82 (3), 561–574. 10.1093/jaoac/82.3.561. 10367374

[ref55] Aadil R. M.; Madni G. M.; Roobab U.; Rahman U; Zeng H-A. Quality control in beverage production: an overview. In Quality control in the beverage industry.; Grumezescu A, Holban A. M., Eds.; Elsevier Inc.: Amsterdam, Netherlands, 2019. 10.1016/B978-0-12-816681-9.00001-1

[ref56] Aznar R. Determination of emerging contaminants in environmental matrices. Doctoral Thesis. Universidad Politécnica de Madrid, Madrid, Spain, 2016.

[ref57] Gross J. H. Mass Spectrometry: a textbook, 3rd ed.; Springer Cham: Heidelberg, Germany, 2017. 10.1007/978-3-319-54398-7

[ref58] Butler J; Conoley M. Application Note 10017; Thermo Electron Corporation, 2005.

[ref59] Tadeo J. L.; Sánchez-Brunete C.; Pérez R. A.; Fernández D. Analysis of herbicide residues in cereals, fruits and vegetables. J. Chromatogr A 2000, 882 (1–2), 175–191. 10.1016/S0021-9673(00)00103-5. 10895942

[ref60] Wong J. W.; Webster M. G.; Bezabeh D. Z.; Hengel M. J.; Ngim K. K.; Krynitsky A. J.; et al. multiresidue determination of pesticides in malt beverages by capillary gas chromatography with mass spectrometry and selected ion monitoring. J. Agric. Food Chem. 2004, 52 (21), 6361–6372. 10.1021/jf040109g. 15478993

[ref61] Navarro S.; Pérez G.; Mena L; Navarro G.; Vela N.; Valverde P. Evolución de residuos de plaguicidas desde el lúpulo (*Humulus lupulus* L.) a la cerveza. Cerveza y Malta. 2005, 166, 26–30.

[ref62] Navarro S.; Vela V. Determinación analítica de residuos de plaguicidas en productos de la industria cervecera. Cerveza y Malta 2006, 172, 26–32.

[ref63] Omote M.; Harayama K.; Sasaki T.; Mochizuki N.; Yamashita H. Analysis of Simultaneous Screening for 277 Pesticides in malt and beer by liquid chromatography with tandem mass spectrometry. J. Am. Soc. Brew Chem. 2006, 64 (3), 139–150. 10.1094/ASBCJ-64-0139.

[ref64] Hengel M. J.; Miller D.; Jordan R. Development and validation of a method for the determination of pesticide residues in beer by liquid chromatography-mass spectrometry. J. Am. Soc. Brew Chem. 2016, 74 (1), 49–52. 10.1094/ASBCJ-2016-1115-01.

[ref65] Bedassa T.; Megersa N.; Gure A. Salting-out Assisted Liquid-Liquid Extraction for the Determination of Multiresidue Pesticides in Alcoholic Beverages by High Performance Liquid Chromatography. Sci. J. Anal Chem. 2017, 5 (3), 38–45. 10.11648/j.sjac.20170503.11.

[ref66] He N. X.; Bayen S. An overview of chemical contaminants and other undesirable chemicals in alcoholic beverages and strategies for analysis. Compr Rev. Food Sci. Food Saf. 2020, 19, 3916–3950. 10.1111/1541-4337.12649. 33337040

[ref67] Pires N. A.; Gonçalves De Oliveira M. L.; Gonçalves J. A.; Faria A. F. Multiclass Analytical Method for Pesticide and Mycotoxin Analysis in Malt, Brewers’ Spent Grain, and Beer: Development, Validation, and Application. J. Agric. Food Chem. 2021, 69 (15), 4533–4541. 10.1021/acs.jafc.0c07004. 33847116

[ref68] Eaton B. An overview of brewing. InHandbook of Brewing; Priest F. G., Stewart G. G., Eds.; Taylor & Francis: Boca Raton, FL, 2006; pp 77–90.

[ref69] Codex Alimentarius Commission (CAC). Pesticide Residues in Food and Feed. Plant Production and Protection Division. http://www.fao.org/fao-who-codexalimentarius/standards/pestres/en (accessed on 2022-08-12).

[ref70] Desmarchelier J. M.; Goldring M.; Horgan R. Predicted and observed residues of bioresmethrin, carbaryl, fenitrothion, d-fenothrin, methacrifos and pirimiphos-methyl on rice and barley after storage, and losses of these insecticides during processing. J. Pestic Sci. 1980, 5 (4), 539–545. 10.1584/jpestics.5.539.

[ref71] Timme G.; Frehse H. Statistical Interpretation and graphic representation of the degradation behaviour of pesticide residues. I. Planzenschutz Nachr Bayer. 1980, 33 (1), 47–60.

[ref72] Timme G.; Frehse G.; Laska V. Statistical interpretation and graphic representation of the degradational behaviour of pesticide residues. II. Planzenschutz Nachr Bayer. 1986, 39 (2), 187–203.

[ref73] Morton R.; Brayan J. G; Desmarchelier J. M; Dilli S.; Haddad P. R; Sharp G. J Statistical analysis of decay of organophosphorus and pyrethroid insecticides and carbaryl on paddy rice, maize, sunflowers and field peas. J. Stored Prod Res. 2001, 37 (3), 277–285. 10.1016/S0022-474X(00)00030-8. 11172864

[ref74] Desmarchelier J. M. A model of the breakdown of dichlorvos on grain. Aust J. Exp Agr. 1977, 17 (88), 818–825. 10.1071/EA9770818.

[ref75] Desmarchelier J. M. Loss of fenitrothion on grain in storage. Pest Sci. 1978, 9 (1), 33–38. 10.1002/ps.2780090107.

[ref76] Bamforth C. W.; Barclay A. H. P. Malting Technology and the Uses of Malt. In Barley: Chemistry and technology; MacGregor A. W., Hatty S. R., Eds.; American Association of Cereal Chemists, Inc.: St. Paul, MN, 1993; pp 297–354.

[ref77] Armitage D. M.; Baxter D. E.; Collins D. A. The fate and efficacy of pesticides applied to malting barley during storage and processing. In Stored malting barley: management of quality using an expert system; Fleurat-Lessard F., Ndiaye A, Knight J. D., Eds.; Institut National de la Recherche Agronomique: Paris, France, 2005; pp 137–165.

[ref78] Miyake Y.; Tajima R.; Ono M. Fate of pesticide metabolites on malt during brewing. J. Am. Soc. Brew Chem. 2003, 61 (1), 33–36. 10.1094/ASBCJ-61-0033.

[ref79] Valverde P. Bagazo y su futuro. Cerveza y Malta 1994, 22, 2–16.

[ref80] Walsh D. B.; O’Neal S. D.; George A. E.; Groenendale D. P.; Henderson R. T.; Groenendale G. M.; Hengel M. J. Evaluation of pesticide residues from conventional, organic, and nontreated hops on conventionally hopped, late-hopped, and wet-hopped beers. J. Am. Soc. Brew Chem. 2016, 74 (1), 53–56. 10.1094/ASBCJ-2016-1115-02.

[ref81] Navarro S.; Vela N.; Pérez G.; Navarro G. Effect of sterol-inhibiting (SBI) fungicides on the fermentation rate and quality of young ale beer. Food Chem. 2011, 126 (2), 623–629. 10.1016/j.foodchem.2010.11.069.

[ref82] Lewis K. A.; Tzilivakis J.; Warner D. J.; Green A. An international database for pesticide risk assessments and management. Hum Ecol Risk Assess. 2016, 22 (4), 1050–1064. 10.1080/10807039.2015.1133242.

[ref83] Pflaum T.; Hausler T.; Baumung C.; Ackermann S.; Kuballa T.; Rehm J.; Lachenmeier D. W. Carcinogenic compounds in alcoholic beverages: An update. Archiv Toxicol. 2016, 90 (10), 2349–2367. 10.1007/s00204-016-1770-3. 27353523

[ref84] Cabras P.; Garau V. L.; Angioni A.; Farris G. A.; Budroni M.; Spanedda L. Interactions during fermentation between pesticides and oenological yeasts producing H_2_S and SO_2_. Appl. Microbiol. Biotechnol. 1995, 43 (2), 370–373. 10.1007/BF00172841.

[ref85] Mackay D.; Shiu W.; Sutherland R. Determination of air-water Henrys Law constants for hydrophobic pollutants. Environ. Sci. Technol. 1979, 13 (3), 333–337. 10.1021/es60151a012.

[ref86] Vanderhaegen B.; Neven H.; Verachtert H.; Derdelinckx G. The chemistry of beer aging-a critical review. Food Chem. 2006, 95 (3), 357–381. 10.1016/j.foodchem.2005.01.006.

[ref87] Cabras P; Meloni M; Pirisi F. M.; Ware G. W. Pesticide fate from vine to wine. Rev. Environ. Contam. Toxicol. 1987, 99, 83–117. 10.1007/978-1-4613-8719-0_4. 3303181

[ref88] Navarro G; Navarro S. Winemaking Problem. Stuck and Sluggish Fermentation. In Concise Encyclopedia of Science and Technology of Wine; Joshi V. K., Ed.; CRC Press: Boca Raton, FL, 2021; pp 297–311.

[ref89] Civille G. V.; Oftedal K. N. Sensory evaluation techniques - make ″good for you″ taste ″good″. Physiol Behav. 2012, 107 (4), 598–605. 10.1016/j.physbeh.2012.04.015. 22554616

[ref90] Batistote M.; Cruz S. H.; Ernandes J. R. Altered patterns of maltose and glucose fermentation by brewing and wine yeasts influenced by the complexity of nitrogen source. J. Inst Brew. 2006, 112 (2), 84–91. 10.1002/j.2050-0416.2006.tb00235.x.

[ref91] Koller W. Sterol demethylation inhibitors: mechanism of action and resistance. In Fungicide resistance in North America; Delp C. J., Ed.; APS Press: St. Paul, MN, 1988; pp 79–88.

[ref92] Buchenauer H. Mechanism of action of triazolyl fungicides and related compounds. In Modern selective fungicides: Properties, applications and mechanism of action; Lyr H, Buchenhauer H, Eds.; Longman Group UK Ltd.: London, UK, 1987; pp 205–231.

[ref93] Piazzon A.; Forte M.; Nardini M. Characterization of phenolics content and antioxidant activity of different beer types. J. Agric. Food Chem. 2010, 58 (19), 10677–10683. 10.1021/jf101975q. 20822144

[ref94] Pérez G; Mena L; Navarro G; Vela N; Navarro S. Book of Abstracts of 6th European Pesticide Residue Workshop; Corfu, Greece, 2006; p 229.

[ref95] Robert T. Metabolic pathways of agrochemicals. Part one: Herbicides and plant growth regulators; The Royal Society of Chemistry: Cambridge, UK, 1998.

[ref96] Robert T; Hutson D. Metabolic pathways of agrochemicals. Part two: Insecticides and fungicides; The Royal Society of Chemistry: Cambridge, UK, 1999.

[ref97] Lesage S. Reduction of the formation of ethylenethiourea from ethylene-bis(dithiocarbamates) by cupric ions in aqueous-media. J. Agric. Food Chem. 1980, 28 (4), 787–790. 10.1021/jf60230a043.

[ref98] Nitz S.; Moza P. N.; Kokabi J.; Freitag D.; Behechti A.; Korte F. Fate of ethylenebis(dithiocarbamates) and their metabolites during the brew process. J. Agric. Food Chem. 1984, 32 (3), 600–603. 10.1021/jf00123a045.

[ref99] Turner J. A. The Pesticide Manual, 17th ed; British Crop Protection Council: Hampshire, UK, 2016.

